# Conditional Deletion of *Pdcd1* Identifies the Cell-Intrinsic Action of PD-1 on Functional CD8 T Cell Subsets for Antitumor Efficacy

**DOI:** 10.3389/fimmu.2021.752348

**Published:** 2021-11-29

**Authors:** Sukanya Raghavan, Nataliya Tovbis-Shifrin, Christina Kochel, Anandi Sawant, Marielle Mello, Manjiri Sathe, Wendy Blumenschein, Eric S. Muise, Alissa Chackerian, Elaine M. Pinheiro, Thomas W. Rosahl, Hervé Luche, Rene de Waal Malefyt

**Affiliations:** ^1^ Department of Immunology, Merck & Co., Inc., Palo Alto, CA, United States; ^2^ Department of Microbiology and Immunology, Institute for Biomedicine, University of Gothenburg, Gothenburg, Sweden; ^3^ Centre d’Immunophénomique - CIPHE (PHENOMIN), Aix Marseille Université (UMS3367), National Institute of Health and Medical Research (INSERM) (US012), The French National Centre for Scientific Research (CNRS) (UMS3367), Marseille, France; ^4^ Merck & Co., Inc., Boston, MA, United States; ^5^ Merck & Co., Inc., Kenilworth, NJ, United States

**Keywords:** conditional KO mice, IFN gamma, PD-1, mass cytometry (CyTOF), tumor immunity and immunotherapy, CD8 T cell

## Abstract

Programmed cell death-1 (PD-1) blockade has a profound effect on the ability of the immune system to eliminate tumors, but many questions remain about the cell types involved and the underlying mechanisms of immune activation. To shed some light on this, the cellular and molecular events following inhibition of PD-1 signaling was investigated in the MC-38 colon carcinoma model using constitutive (PD-1 KO) and conditional (PD1cKO) mice and in wild-type mice treated with PD-1 antibody. The impact on both tumor growth and the development of tumor immunity was assessed. In the PD-1cKO mice, a complete deletion of *Pdcd1* in tumor-infiltrating T cells (TILs) after tamoxifen treatment led to the inhibition of tumor growth of both small and large tumors. Extensive immune phenotypic analysis of the TILs by flow and mass cytometry identified 20-different T cell subsets of which specifically 5-CD8 positive ones expanded in all three models after PD-1 blockade. All five subsets expressed granzyme B and interferon gamma (IFNγ). Gene expression analysis of the tumor further supported the phenotypic analysis in both PD-1cKO- and PD-1 Ab-treated mice and showed an upregulation of pathways related to CD4 and CD8 T-cell activation, enhanced signaling through costimulatory molecules and IFNγ, and non-T-cell processes. Altogether, using PD-1cKO mice, we define the intrinsic nature of PD-1 suppression of CD8 T-cell responses in tumor immunity.

## Introduction

Immune therapy with antibodies to programmed cell death-1 (PD-1)/programmed death-ligand 1 (PD-L1) (PD-1 blockade) has been approved for treatment of patients with a growing list of cancers including malignant metastatic melanoma, non-small cell lung cancer, head and neck and esophageal squamous cell carcinoma, renal cell carcinoma, Hodgkin’s lymphoma, bladder cancer, cervical cancer, gastric cancer, hepatocellular carcinoma, Merkel cell carcinoma, and solid tumors with microsatellite instability. Many ongoing clinical trials are evaluating PD-1 blockade across additional tumor types alone or in combination with chemotherapy or other checkpoint inhibitors (1). In spite of the success of PD-1 blockade to enhance overall survival (OS) in multiple tumor types compared to chemo- or radiation therapy, primary resistance to immune checkpoint inhibitors can occur and counteract the actual clinical benefits. Furthermore, after an initial response to PD-1 blockade, acquired resistance can also occur in some patients, which often leads to disease progression or eventually relapse. The mechanisms of resistance are complex, including both tumor cell intrinsic and extrinsic factors (2). In the current study, the immune cell phenotypes and gene expression pathways necessary for tumor regression after PD-1 blockade were investigated. The findings from this study can contribute to the improvement of current PD-1 blockade therapies for non-responding patients and develop future combination strategies aimed at targeting the immune system.

*Pdcd1* gene knockout mice (PD-1KO) have been useful to elucidate the function of PD-1 in central and peripheral tolerance and development of autoimmunity (3). Lack of PD-1 from birth affects the immune system, particularly the T-cell populations. In the peripheral lymphoid organs of PD-1KO mice, a significant increase in the baseline frequency of effector memory CD4^+^ and CD8^+^ T cells (CD44^+^CD62L^−^) has been reported (3, 4). Immune-mediated mechanisms prior to tumor rejection have been difficult to follow in the PD-1KO mice, as the tumors in some mice fail to establish or quickly regress (5). The availability of PD-1 neutralizing antibodies has made it possible to define the effects of PD-1 blockade on the immune system without the confounding effects of lacking PD-1 from birth. However, antibody treatment can have unintended consequences that could affect mechanism of action studies. Besides the potential to develop anti-drug antibody responses or Fc–FcR interactions that may lead to immune modulation that can be controlled, another mechanism that can interfere with efficacy of PD-1 antibodies has been described. Macrophages were demonstrated to capture and remove the PD-1Ab bound on CD8^+^ T cells through FcγRIIB/III interactions making the PD-1 on the CD8^+^ T cells, now sensitive to the interaction with PD-L1 and partial tumor regression (6). This could be overcome by administration of FcR blocking antibodies in combination with PD-1 Ab to tumor-bearing mice, which then led to complete tumor regression in all mice. Thus, studies on mechanisms of tumor immunity by PD-1 blockade using constitutive gene knockout mice or Ab treatment should consider these limitations.

Nevertheless, studies in PD-1KO and PD-1 Ab-treated mice have provided many insights into the biology of PD-1 and immune mechanisms leading to tumor growth or shrinkage (7, 8). An immune-PET method for monitoring tumor-infiltrating cells during tumor growth showed a complete response to PD-1 Ab treatment only in tumors fully infiltrated by CD8^+^ T cells. Shrinkage of tumors after complete responses also correlated with an increase in the CD11b^+^ macrophage population in the center of the tumors with an M1-like signature (9). The efficacy of PD-1 immune therapy has also been shown to depend on interferon gamma (IFNγ) sensing by tumor cells in the tumor microenvironment, which can be regulated by tyrosine-protein phosphatase non-receptor type 2 (*Ptpn2*) (10, 11). Although IFNγ upregulates major histocompatibility complex (MHC) expression and antigen presentation on myeloid cells, in the tumor microenvironment, it is indeed one of the most potent drivers of PD-L1 expression on immune cells and tumor, which can counteract the effects of PD-L1 blocking antibodies, leading to “adaptive resistance” (8, 12).

Conditional gene knockout mice in which a specific genetic deletion can be induced in adult mice can overcome some of the weaknesses of the currently available PD-1KO and PD-1 Ab-treated models to study the function of PD-1 in tumor immunity (13). However, a direct comparison to this effect is lacking. The only reported conditional gene knockout of a checkpoint receptor in the literature to date is the CTLA-4 conditional knockout mice (14, 15). The loss of *ctla-4* in CD4^+^ T cells and Foxp3^+^ regulatory T cells was found to expand not only the effector T cells but also of CD4^+^Foxp3^+^ regulatory T cells, leading to new and unexpected insights into the function of CTLA-4 in peripheral tolerance, development of autoimmunity, and tumor immunity. Likewise, to understand the function of PD-1 in tumor immunity without the compensatory effects of lacking *Pdcd1* from birth, we established a Rosa26cre^ERT2^PD-1^fl/fl^ (PD-1 conditional knockout) model genetically engineered to induce *Pdcd1* deletion in adult mice by tamoxifen treatment.

In the current study, the growth of tumor cells explored after three methods of PD-1 blockade was found to show a range of response, namely, complete inhibition, partial inhibition of tumor growth, and escape. Long-term memory immune response induced in the mice with complete inhibition of tumor growth was found to be antigen specific. Comprehensive flow and mass cytometry profiling of the tumor-infiltrating lymphocytes prior to tumor growth inhibition identified the infiltration of 20 different immune cell subsets, of which 5 of the CD8 T subsets expanded specifically after PD-1 blockade. In the large tumors after deletion of *pdcd1* in PD-1cKO mice, an immunogenic cell signature was identified with cytotoxic CD8 T cells expressing IFNγ and effector memory CD4 T cells expressing granzyme B. Bulk RNA sequencing of the tumors from PD-1cKO and PD-1 Ab-treated mice revealed 2,184 genes to be commonly upregulated including *cd8a*, *tbx21*, *klrc1*, and *ifng*, and the top 10 highly significantly affected pathways related to immune cell infiltration and signaling in immune cells. Altogether, our study underpins the importance of functional CD8^+^ T cells in tumor immunity during PD-1 blockade, eliminates any doubts about effector or off-target effects of antibody treatment for anti-tumor efficacy, and advances the knowledge of specific TIL subpopulations prior to tumor growth inhibition, with the potential to be biomarkers of clinical response.

## Materials and Methods

### Generation of *Pdcd1* Constitutive KO Mice by CRISPR/Cas9-Mediated Gene Editing

PD-1KO mice were generated at Taconic Biosciences Inc. (16). The targeting strategy was based on the National Center for Biotechnology Information (NCBI) transcript NM_008798_2 and involved deletion of exons 2 and 3 resulting in in-frame splicing from exon 1 to exon 4 and expression of a PD-1 protein lacking ligand binding and transmembrane domains. A similar approach was successfully used by Keir et al. (17). Off-target prediction was performed to identify potential single guide RNAs (sgRNAs) with the lowest number of predicted off-target mutations (18) and 5′-CT**ATATACCCGACCGCAGGTTC**AA-3′ (proximal sgRNA) and 5′-CA**GGAACTCCCCGTTAGTAAAT**GG-3′ (distal sgRNA) selected. Cas9 mRNA and sgRNAs were coinjected into C57BL/6NTac zygotes to delete exons 2 and 3 by Cas9-mediated genome editing and implanted in pseudo-pregnant females. Nine founder animals were identified for genomic deletions by PCR analysis spanning the junction site. Six of these animals were further characterized by subcloning and sequencing of the 170-bp PCR DNA fragments. All six animals carried the genomic deletion of exons 2 and 3 with two animals having perfect end joining. PD-1KO and WT control mice were established from one of the founders, which had no deletions at the junction.

### Generation of *Pdcd1* Conditional KO Mice

PD-1cKO mice were also generated at Taconic Biosciences Inc. The PD-1cKO mouse was generated by homologous recombination with a targeting vector in which loxP sequences flanked exons 2 and 3 of *Pdcd1*. Puromycin resistance, the positive selection marker for loxP insertion, was inserted into intron 1 flanked by FRT sites. The final targeting vector was generated using genomic DNA from BAC clones from the C57BL/6J RPCIB-731 BAC library and transfected into a C57BL/6N Tac ES cell line. Homologous recombinant ES cell clones were isolated using positive selection. After *in vivo* Flp-mediated removal of the selection marker gene, we obtained the conditional KO allele. These PD-1cKO (Pdcd1^fl/fl^) mice were then further bred with Rosa26-creERT2 mice (19) to generate homozygous PD-1cKO mice with creERT2 (ROSA26^creERT2^Pdcd1^fl/fl^) and homozygous PD-1cKO mice without the creERT2 (Pdcd1^fl/fl^).

### Mice

Age-matched 8–10-week-old female wild-type C57/BL6NTac (WT), PD-1KO, PD-1cKO, and respective littermate wild-type mice were bred at the Taconic Biosciences Inc. facility in Germantown, NY, under specific pathogen-free conditions. All animal procedures were approved by the Institutional Animal Care and Use Committee (IACUC) of Merck Research Laboratories (APS# 400265), in accordance with guidelines of the Association for Assessment and Accreditation of Laboratory Animal Care.

### Mouse Cell Lines, Tumor Injection, and Measurement

#### For Flow Cytometry

MC-38 mouse colon carcinoma cell line was obtained from the National Cancer Institute. The mouse melanoma cell line B16F10 (ATCC) was also used. Cell lines were expanded and maintained in complete Dulbecco modified essential medium containing glutamine and sodium pyruvate (Corning, Celgro), supplemented with 10% fetal bovine serum (GE Healthcare, South Logan, UT) and penicillin-streptomycin (Gibco, Life technologies; 100 U/ml). Cell lines stored in liquid nitrogen were thawed, cultured in flasks, and passaged before injection to mice. Prior to implantation, cells were counted and viability determined (typically >90%). Mice were subcutaneously injected on the right flank with 1 × 10^6^ cells/mouse. The tumor growth was measured in individual mice using electronic calipers, twice a week for a period of 4–6 weeks. The tumor volume was calculated using the formula: length × width × width/2 (mm^3^). Mice were randomized before start of treatment when tumors reached approximately 100 mm^3^ (range, 100–120 mm^3^). In the PD-1 Ab-treated, PD-1KO, and PD-1cKO mice that previously showed complete response to primary MC-38 injection, a rechallenge of MC-38 tumor (1–2 × 10^6^) or B16F10 (0.1 × 10^6^) injection was performed 4 weeks later with naive age-matched WT mice also injected at the same time as controls. The tumor growth was measured in individual mice as described earlier, twice a week for a period of 4–6 weeks. Lymph nodes were isolated from the mice 2–13 days post-tumor challenge or rechallenge in PD-1cKO and WT controls, and flow cytometry analysis of different immune cell populations was carried out.

#### For Mass Cytometry

Female 8–10-week-old mice were injected subcutaneously on the right flank with MC-38 cells (0.5 × 10^6^). Mice were weighed twice a week and tumor measured with electronic calipers three times a week for a period of 3–4 weeks (13 days for rechallenge experiment). When the tumor volume reached 50–100 mm^3^, the mice were randomized to different groups.

### PD-1 Antibody or Tamoxifen Treatment

WT mice were randomized day 7 post-tumor implant, and for PD-1 antibody (Ab) experiments, mice were dosed intraperitoneally every 4 days on four separate occasions with 5 mg/kg antimouse PD-1 Ab muDX400 (16). PD-1cKO mice were randomized day 7 post-tumor implant, and tamoxifen (Sigma-Aldrich) dissolved in 1:9 ratio of ethanol:sunflower oil at a stock concentration of 10 mg/ml was injected intraperitoneally at a dose of 40 mg/kg on 5 consecutive days. Tamoxifen injections were administered to both PD-1cKO and littermate controls.

### Isolation of Tissue-Infiltrating Lymphocytes

#### For Flow Cytometry

Tumors from PD-1 Ab-treated (day 15 post-tumor implant), PD-1KO (day 11 post-tumor implant), and PD-1cKO mice (day 11, 15, and 22 post-tumor implant) and respective WT controls were excised and weighed. The tumors were mechanically disrupted using the Gentle MACS (Miltenyi Biotech) to obtain a single-cell suspension of tumor-infiltrating lymphocytes (TILs). The cells were filtered and counted before staining and flow cytometry acquisition and analysis.

#### For Mass Cytometry

The tumors from PD-1 Ab-treated, PD-1KO, and PD-1cKO (day 18 post-tumor implant) mice and respective WT controls were collected and digested using the tumor dissociation kit (Miltenyi Biotech), followed by isolation of CD45+ TILs using the CD45 (TIL) MicroBead kit (Miltenyi Biotech). The cells were counted before proceeding for cell surface staining.

### Antibodies

The antibodies used for flow and mass cytometry staining are listed in **Supplementary Table S1**.

### Antibody Staining and Data Acquisition

#### Flow Cytometry

Single cell suspension of TILs and spleen was stained with the live dead exclusion stain ef506 (eBioscience). The cells were subsequently incubated in Fc block (CD16/32) (BD Biosciences) for 30 min before proceeding with surface staining with fluorescently conjugated antibodies (**Supplementary Table S1**). Intracellular staining with fluorescently conjugated antibodies was carried out after permeabilization using the Foxp3 staining buffers according to the manufacturer’s instruction (eBioscience). The samples were acquired on the same day on the LSR Fortessa X-20 (BD Biosciences), and the data were analyzed using the FlowJo software (v10.0).

#### Mass Cytometry

The tumors were collected from mice and digested using the tumor dissociation kit (Miltenyi Biotech), followed by isolation of CD45^+^ TILs using the CD45 (TIL) MicroBead kit (Miltenyi Biotech). Staining of cells with CisPt 194 was followed by incubation with Fc block and, subsequently, the panel of antibodies for cell surface markers. To perform intracellular staining, the fixation/permeabilization buffer was used (BD Biosciences). The cells were permeabilized for 30 mins at 4°C and stored overnight in the cell staining medium, and subsequently, bar coding was performed using the barcoding reagent according to manufacturer’s instructions (Fluidigm). Next, intracellular staining for cytokines or transcription factors was performed by incubating the cells in the respective antibody mixture. Finally, the cells were incubated overnight in permeabilization buffer and iridium nucleic acid intercalator (Fluidigm). Prior to acquisition, cells were washed and then diluted to 5 × 10^6^ cells/ml in dH_2_O containing 10% (v/v) EQ Four Element Calibration Beads (Fluidigm) and filtered. Cells were acquired at a rate of 400 cells/s using a Helios mass cytometer (Fluidigm). Flow Cytometry Standard (FCS) files were normalized to EQ bead signal and debarcoded using Fluidigm de barcoder algorithm. Files were analyzed using Cytobank CytoF v6.7 (Fluidigm).

### Bulk Tumor RNA Preparation, Sequencing, and Data Analysis

WT mice were subcutaneously injected in the right flank with MC-38 cells (1 × 10^6^) and received PD-1 Ab injection day 7, 11, and day 15 post-tumor implant. PD-1cKO and littermate control mice were subcutaneously injected in the right flank with MC-38 cells (1 × 10^6^), and day 6–7 post-tumor cell injection, the mice received injections of tamoxifen on 5 consecutive days. On days 11 and 15 for the PD-1 Ab-treated mice, or day 22 (PD-1cKO) post-tumor implant, tumors were excised and snap frozen in liquid nitrogen and stored at −80°C until RNA isolation. For tumor tissue RNA isolation, organs were homogenized into RNA STAT-60 (Tel-Test Inc., Friendswood, TX) using a polytron homogenizer; then, total RNA was extracted according to the manufacturer’s instructions. After isopropanol precipitation, total RNA was re-extracted with phenol:chloroform:isoamyl alcohol (25:24:1) (Sigma-Aldrich, St. Louis, MO). Sequencing was performed using the Truseq stranded total RNA RiboZero library preparation kit (Catalog no. RS-122-2201) according to the manufacturer’s instructions (Illumina, San Diego, CA). The resulting cDNA libraries were sequenced on an Illumina (HiSeqTM 4000) using a 50-base paired-end run. Alignment and differential gene expression analysis was performed in Omicsoft Array Studio (Qiagen, Germantown, MD) version 10.0.1.118. Briefly, cleaned reads were aligned to the Mouse.B38 genome reference using the Omicsoft Aligner with a maximum of two allowed mismatches. Gene level raw counts, and fragments per kilobase of transcript per million mapped reads (FPKM), were determined by the OSA algorithm as implemented in Omicsoft Array Studio and using Ensembl.R86 gene models. Approximately 90% of reads across all samples mapped to the reference genome (corresponding to between 40 and 160 million reads). Normalization of gene counts and differential expression analysis were performed based on modeling the raw counts within the framework of a negative binomial model using the R package DESeq2 (v1.22.2) yielding fold change and corrected p-values (false discovery rate, Benjamini–Hochberg; FDR_BH). Samples from vehicle-treated, and wild-type, animals were used as baseline for PD-1 Ab-treated, and PD-1cKO, comparisons, respectively. Gene signatures were identified by applying a cutoff of 1.2-, 1.5-, or 2-fold change, adjusted p-value (FDR) <0.01, and a normalized count > 20 in either of the two treatment groups within the comparison. Genes unique to one treatment were identified by first applying the above cutoffs to the first treatment, then applying more stringent cutoffs for the second treatment [absolute fold change <1.2, nominal and adjusted p value (FDR) >0.1]. Heatmaps were created using Omicsoft Array Studio. The log2 fold change in gene expression in PD-1 Ab-treated and PD-1cKO compared to isotype-injected or WT mice, respectively, was calculated and the ingenuity pathway analysis utilized to explore the canonical pathways significantly enriched in the data set. The Broad Institute Gene Set Enrichment Analysis software (20) was used to run analyses on normalized gene expression data with prefiltering for low counts. The c2_all, c2_cp, and the hallmarks gene set database was included in the analysis. A total of 1,000 permutations were performed. Gene sets enriched at a nominal p < 0.05 and FDR < 0.25 were considered significant (GEO accession number GSE171274).

### Mass Cytometry Analysis

Mass cytometry analysis was performed as previously described (21). Briefly, the FCS files generated from mass cytometry were manually gated to live CD45+ cells using Cytobank. Each sample were merged by the experimental group for subsequent analysis. Preprocessing of the raw data was followed by dimensionality reduction and visualization by t-distributed stochastic neighbor embedding (t-SNE) using the default parameters (perplexity = 30 and iterations = 1,000). PhenoGraph (21) was performed using in-house developed R-shiny interface “CIPHEBox” to classify and visualize the subpopulations of tumor-infiltrating lymphocytes (TILs) based on the cell surface and intracellular markers listed in the **Supplementary Table S1**. PhenoGraph first identified the k-nearest neighbors (k = 30) using Euclidean distance and calculated the similarities using the Jaccard coefficient. Subsequently, the Louvain algorithm was used to partition the network for detecting communities with optimal modularity, generating 21 metaclusters. Expert-guided manual annotation of metaclusters was performed using a heatmap of median expression values of the initial automated 21 R-Phenograph metacluster and t-SNE overlaid marker expression values. When comparing different experimental groups, the t-SNE and clustering analyses were performed on the combined datasets. Force- and landmark- directed maps were generated with a modified version of the Scaffold application (22) and the 21 landmark populations that were defined by Phenograph. A built-in clustering algorithm Clara was used to generate 200 nodes as unsupervised scaffold clusters. This allowed cross-experiment comparison of different CyTOF datasets using metaclusters-guided manual metaclustering by the grouping of Clara nodes around phenograph metacluster landmarks validated and annotated by an expert in the Scaffoldmap. Data are presented as mean with SD unless otherwise stated. FC Radar plot was used to visualize variations of leukocyte proportions between the PD-1 mutants (PD-1KO and PD-1cKO), PD-1Ab-treated and control animals.

### Statistical Analysis

#### Tumor Volume

Comparisons of changes in tumor volume between treatments were made at each day of follow-up, and collectively over all time points using area under the curve (AUC) as a summary measure for each tumor. To compare two treatment groups on a given day, the Peto and Peto version of the Gehan–Breslow test for right-censored data was used (23). In the absence of censoring, this reduces to the non-parametric Mann–Whitney (or Wilcoxon rank sum) test. Two-sided p-values were estimated from 20,000 random reassignments of animals between the two treatments being compared. To control the familywise error rate across all time points for a given pair of treatments, p-values were multiplicity adjusted by Holm’s method. A p-value of <0.05 was used to define statistical significance.

#### Flow Cytometry

Mann-Whitney test or ANOVA with Dunnett’s multiple comparison tests was used for comparison frequencies of cells assessed by flow cytometry staining in PD-1 Ab-treated, PD-1KO, or PD-1cKO mice or compared to the control group. Statistical analysis was performed using Prism ver. 7.0 GraphPad Software, La Jolla, CA).

#### Mass Cytometry

Mean expression intensities of markers for all subpopulations on all mice of the study were pooled in one single spreadsheet per R-phenograph cluster per organ, transformed in asinh and centered to the mean. The resulting list was run in the SIMCA16 multivariate analysis software (Sartorius). Orthogonal partial least square-discriminant analysis (OPLS-DA) method was applied on the dataset to identify groups of samples presenting similar types of variations. Variable importance in projection (VIP) was selected with a VIP value over 1. A list of parameters with VIP >1 was subjected to reduction. Predictive variables with VIP >1 were uploaded into TMeV, a microarray software suite, in order to build a heatmap after median centering and SD reduction. Dataset was clustered using Euclidian distance on samples and variables by hierarchical clustering. All independent values were visualized with a color code spanning values of −1 to +1.

## Results

### Deletion of PD-1 in Adult Mice Results in Regression of MC-38 Tumors, Immunity, and Long-Term Antigen-Specific Memory

MC-38 colon carcinoma cells transplanted in C57BL/6 mice are highly immunogenic and express high levels of PD-L1. Furthermore, a high frequency of the CD4^+^ and CD8^+^ tumor-infiltrating lymphocytes (TILs) express PD-1. The effects of PD-1 on tumor growth were investigated by comparing PD-1 Ab treatment in WT mice to tumor growth in PD-1 constitutive knockout mice (PD-1KO) and PD-1 conditional knockout mice (PD-1cKO) (**Supplementary Figures S1A, B**). Administration of Tamoxifen to induce cre-mediated deletion of *pdcd1* led to a decrease in PD-1 expression on CD4^+^ and CD8^+^ TILs by day 4 post-randomization (day 11 post-implant) in PD-1cKO, mice and this was further reduced at day 9 (day 15 post-implant). At day 15 (day 22 post-implant), <2% of the CD4^+^ or CD8^+^ TILs expressed PD-1 compared to 60% and 85% of CD4^+^ and CD8^+^ T cells respectively expressing PD-1 in WT mice (**Supplementary Figures S1C, D**).

**Figure 1:**
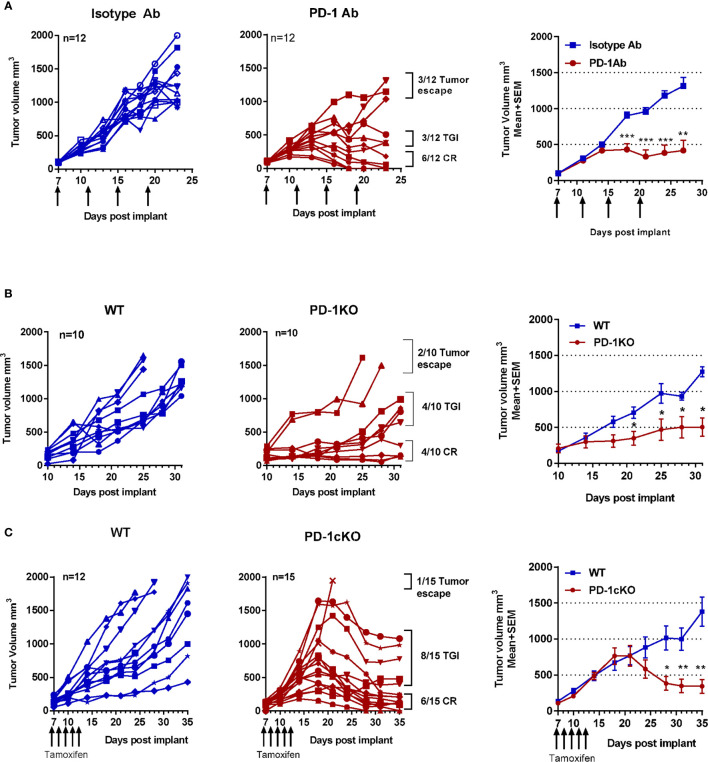
Deletion of PD1 in adult mice results in regression of MC-38 tumors, immunity, and long-term antigen-specific memory. MC-38 tumor size plotted for individual mice and as mean ± SEM for the group. **(A)** WT mice after treatment with PD-1 or isotype antibody. **(B)** PD-1KO and WT control mice. **(C)** PD-1cKO mice and littermate controls after treatment with tamoxifen. Arrows indicate time point of PD-1 Ab or tamoxifen treatment. Data representative of three independent experiments with n = 7–8 mice per experiment and group. *p < 0.05, **p < 0.01, ***p < 0.001. TGI, tumor growth inhibition; CR, complete response. See also **Supplementary Figure S1**.

PD-1 blockade showed a range of responses in tumor-bearing mice, and a pattern did emerge with mice showing either a complete response (CR) (<200 mm^3^), tumor growth inhibition (TGI) (200–1,000 mm^3^), or tumor escape (>2,000 mm^3^) (**Figures 1A**–**C**). The area under curve and the mean tumor size difference after PD-1 blockade were statistically significant in PD-1 Ab-treated, PD-1cKO, and the PD-1KO mice compared to controls (**Figures 1A**–**C**). Noteworthy was the cell intrinsic effect of *Pdcd1* deletion on the tumor growth in adult PD-1cKO mice with a twofold reduction in the size of large tumors within a 7-day period, from day 18–25 post-tumor implant (**Figure 1C**).

All mice with a CR after PD-1 blockade completely rejected a rechallenge with MC-38 cells (secondary injection of cells and tumor growth) (**Supplementary Figure S1E**). In contrast, tumors grew exponentially in naive age-matched WT mice. PD-1 Ab-treated mice with CR were rechallenged with the unrelated but syngeneic B16/F10 melanoma cells. The B16/F10 tumors were established in all challenged mice and grew uninhibited (**Supplementary Figure S1E**), indicating that the memory response was antigen specific in the PD-1 Ab-treated mice. To further characterize the systemic immune activation after challenge and re-challenge of mice with tumor cells, the total immune cells in the draining lymph nodes were counted (**Supplementary Figure S1F**), and phenotypic analysis of CD4 (naive, CM, EM), CD8 (naive, CM, EM), and NK and NKT cell populations was performed (**Supplementary Figure S1G**). In WT mice, tumor growth resulted in an expansion of EM CD8 T cells (CD44^+^CD62L^−^) in the lymph nodes at day 13 post-tumor cell challenge. In the tamoxifen-treated PD-1cKO mice, a reduction in the frequency of the EM CD8^+^ T cells in the lymph nodes was observed compared to tamoxifen-treated WT mice. Finally, in PD-1cKO mice with CR, injection of tumor cells resulted in an expansion of the EM CD8^+^ and CD4^+^ CM and EM T cells in the lymph nodes at day 3 post-tumor cell challenge (**Supplementary Figure S1G**). In summary, PD-1 blockade by antibody treatment or constitutive or conditional *Pdcd1* deletion resulted in not only CR but also TGI and escape in some mice. CR after inhibition of PD-1 was associated with the development of a memory immune response that was also antigen specific. Finally, since the inhibition of tumor growth in the PD-1cKO mice clearly coincided with the deletion of *Pdcd1*, it indicates a cell-intrinsic effect of PD-1 signaling in the TILs.

### Absence of PD-1 Signaling Leads to an Increase in Frequency CD8^+^ICOS^+^ TILs and a Reduction in CD4^+^FoxP3^+^ Tregs

**Figure 2:**
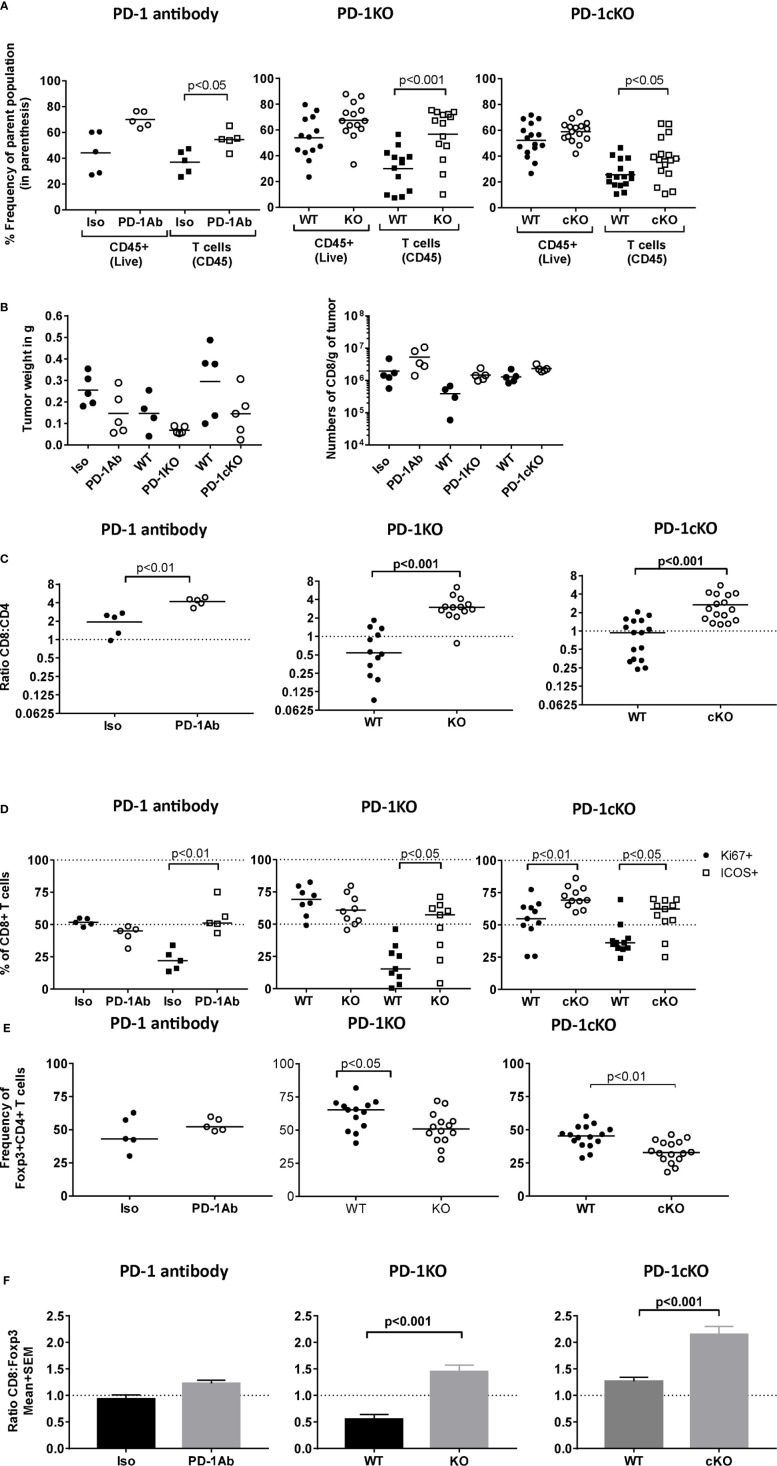
Absence of PD-1 signaling leads to an increase in frequency CD8^+^ICOS^+^ TILs and a reduction in CD4^+^FoxP3^+^ Tregs. Tumors were excised and weighed and tumor-infiltrating lymphocytes were isolated from PD-1 Ab-treated (day 15 post-tumor implant), PD-1KO (day 11 post-tumor implant), and PD-1cKO (day 22 post-tumor implant) mice and multiparameter flow cytometry analysis performed. **(A)** Frequency of CD45^+^ leukocytes of live and frequency of CD3^+^T cells of tumor-infiltrating lymphocytes in PD-1 Ab-treated, PD-1KO, and PD-1cKO mice and their respective controls. **(B)** Weight of tumor in grams and number of cells normalized by tumor size isolated from the tumor of PD-1 Ab-treated, PD-1KO, and PD-1cKO mice and respective control mice. **(C)** Ratio of the frequency of CD8 and CD4 TILs. **(D)** Frequency of Ki67^+^ and ICOS+CD8^+^ TILs. **(E)** Frequency of Foxp3^+^ T cells among the CD4^+^ TILs. **(F)** Ratio of CD8^+^ to Foxp3^+^ T cells in the tumor tissue. Data representative of three independent experiments with n = 4–5 mice/experiment, group, and time point. See also **Supplementary Figure S2**.

To define the immune mechanisms that contribute to tumor immunity after PD-1 blockade, an in-depth characterization of the TILs by flow cytometry was performed that were isolated from PD-1 Ab-treated, PD-1KO, and PD-1cKO mice. The time points of analyses were optimized to capture the ongoing antitumor response at the physiologically equivalent stages in the three different models. A significant increase in the frequency of CD3+T cells isolated from tumors of PD-1-Ab treated, PD-1KO, and PD-1cKO mice compared to the respective control mice was observed but not in the frequency of CD45^+^ cells (**Figure 2A**). An increase in tumor mass or T-cell numbers per tumor mass did not reach statistical significance (**Figure 2B**). In addition to the increased frequency of T cells in the tumors after PD-1 blockade, the ratio of CD8:CD4 T cell subsets was significantly increased (**Figure 2C**). In the PD-1cKO mice, this was first apparent at day 15 post-randomization (22 days post-implant) (**Supplementary Figure S2B**). Proliferation of CD8^+^ TILs most likely contributed to the increased CD8:CD4 ratio in the PD-1cKO as the frequency of CD8^+^Ki67^+^, but not that of CD4^+^Ki67^+^ TILs was increased in the PD-1cKO mice (**Figure 2D** and **Supplementary Figures S2A, C, D**). However, proliferation did not contribute to the increase in frequency of CD8^+^ T cells in the PD-1KO and PD-1 Ab treated mice (**Figure 2D**). PD-1 expressing Foxp3^+^CD4^+^ regulatory T cells in the tumor can block the function of effector T cells, and therefore, it was important to define the effects of regulatory T cells on tumor growth and immunity. Deletion of *Pdcd1* led to a reduction in the frequency of CD4^+^Foxp3^+^ T cells in the PD-1KO and PD-1cKO but not in the antibody-treated mice (**Figure 2E** and **Supplementary Figure S2A**). The increase in frequency of CD8^+^ T cells and decrease in the frequency of Foxp3^+^ cells led to an increase in the ratio of CD8:Foxp3 in the tumors of PD-1KO and PD-1cKO mice (**Figure 2F**).

The TILs were further phenotypically characterized for their expression of checkpoint inhibitors, transcription factors, and activation markers. The activation marker ICOS was specifically upregulated on CD8^+^ (**Figure 2D** and **Supplementary Figure S2A**) but not CD4^+^ T cells (data not shown) in the absence of PD-1 signaling. No changes in the expression of TIGIT, Lag-3, and CTLA-4 on either Foxp3^+^ or Foxp3^−^CD4^+^ T cells or on CD8^+^ T cells were observed in PD-1cKO compared to littermate controls treated with tamoxifen at any of the time points studied (**Supplementary Figures S2C, D**). However, an increased percentage of CD4^+^Foxp3^−^ T cells expressed CTLA-4 was observed at 15 days post-randomization (22 days post-implant) indicating CD4^+^ T-cell activation (**Supplementary Figures S2A, C**). No differences in expression of activation markers CD69 or CD25 was evident on the CD4 and CD8^+^ T cells from PD-1cKO mice versus controls for any of the time points studied (**Supplementary Figures S2C, D**). Finally, no difference was observed in the T-cell immune cell compartments in the spleens of tumor-bearing mice after PD-1 blockade (data not shown). Altogether, tumor growth inhibition after PD-1 blockade was due to the expansion of CD8^+^ T cells expressing the costimulatory molecule ICOS and a concomitant reduction in the frequency of CD4^+^Foxp3^+^ regulatory T cells.

### Absence of PD-1 Signaling Leads Specifically to the Expansion of Activated CD8^+^ Effector Memory Tumor-Infiltrating Lymphocytes

To examine the effector molecules important for tumor growth inhibition under conditions of PD-1 blockade, a mass cytometry T-cell panel including markers for lineage, activation, checkpoint receptors, transcription factors, and cytokine secretion was established (**Supplementary Table S1**).

**Figure 3:**
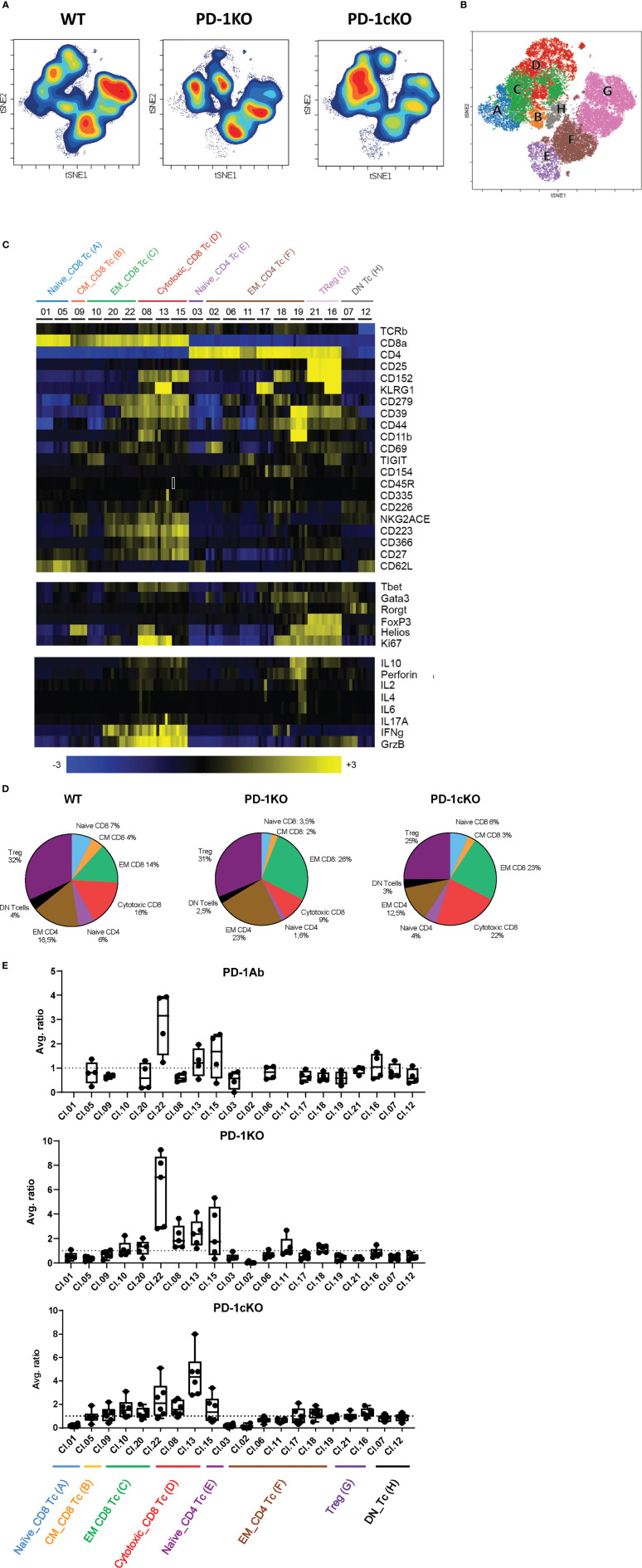
Absence of PD-1 signaling leads specifically to the expansion of activated CD8^+^ effector memory tumor-infiltrating lymphocytes. **(A)** Density tSNE plots of tumor-infiltrating lymphocytes isolated from tumors injected to WT, PD-1KO, or PD-1cKO mice (all 18 days post-implant). **(B)** Projection of the annotation of combined clusters at the single cell level on a tSNE plot. **(C)** Heatmap displaying marker expression in the designated T-cell clusters in WT mice. **(D)** Pie chart displaying the different T cell subsets among the tumor-infiltrating lymphocytes in WT, PD-1KO, or PD-1cKO mice. **(E)** Average ratio of the frequency of the T cell found in each R-phonograph cluster per mouse (mean + SD). The T-cell clusters labeled by their name as in **(C)**. Data representative of two independent experiments with n = 3–5 mice/experiment, group, and time point.

T-cell populations affected by PD-1 blockade (PD-1KO or PD-1cKO) were identified by a data-driven unsupervised clustering approach using R-PhenoGraph as described in *Materials and Methods*. tSNE plots show a dramatic effect of PD-1 blockade on the density but not the phenotype of the tumor-infiltrating T-cell clusters in the PD-1KO and PD-1cKO mice compared to respective control mice (**Figure 3A**). Based on expression of the cell surface and intracellular markers described above, the CD4 and CD8 T cells separated into 8 different cell types and 20 individual clusters. Annotations of cluster combinations were then projected onto a tSNE plot (**Figure 3B**) or as a heat map (**Figure 3C**). Each cluster was assigned a nomenclature related to its function as naive CD4^+^ (cluster 3) and CD8^+^ T cells (cluster 01 and 05), cytotoxic CD8^+^ T cells (cluster 08, 13, and 15), effector memory CD4^+^ (cluster 02, 06, 11, 17, 18, and 19), effector memory CD8^+^ T cells (cluster 10, 20, and 22), central memory CD8^+^ T cells (cluster 09), CD4^+^ regulatory T cells (cluster 16 and 21), and CD4 CD8 double-negative T cells (cluster 07 and 12). The average of the frequencies of the major populations (CD4 and CD8 T-cell EM and CM, cytotoxic T cells, double-negative T cells, and CD4^+^ Treg) were plotted in a pie chart to obtain an overview of the effects of PD-1 blockade on these populations (**Figure 3D**). In line with data presented in **Figure 2**, an increase in the frequency of CD8^+^ T cells in the absence of PD-1 signaling was observed in the PD-1KO and to a lesser extent in PD-1cKO mice (**Figure 3D**).

Further analysis of the immune cell clusters revealed increased ratios of several clusters particularly the effector memory and cytotoxic CD8^+^ T-cell populations (**Figure 3E**). For cluster 22, an average three- and sixfold increase was observed in the tumors from PD-1Ab and PD-1KO mice respectively compared to WT mice. For cluster 22, the cells were defined through the expression of CD8^+^, CD39^+^, CD223^+^ (Lag-3), CD62L^−^, GrzB^+^, and IFNγ, indicating activated CD8^+^ T cells capable of tumor killing function. In the PD1-cKO mice, however, cytotoxic CD8^+^ T cells (cluster 13) was the dominant upregulated cluster, sharing all the markers with cluster 22 and additionally expressing KLRG1, CD27, and Ki67 (**Figure 3E**). The relative expansion of specific CD8 T cells in the PD-1 Ab-treated, PD-1KO, and PD-1cKO compared to respective control mice prior to tumor growth inhibition suggests that it plays an important role in the control of tumor growth in the absence of PD-1 signaling.

### Large and Small Tumors Have a Distinct Phenotypic Signature in the PD-1cKO Mice

**Figure 4:**
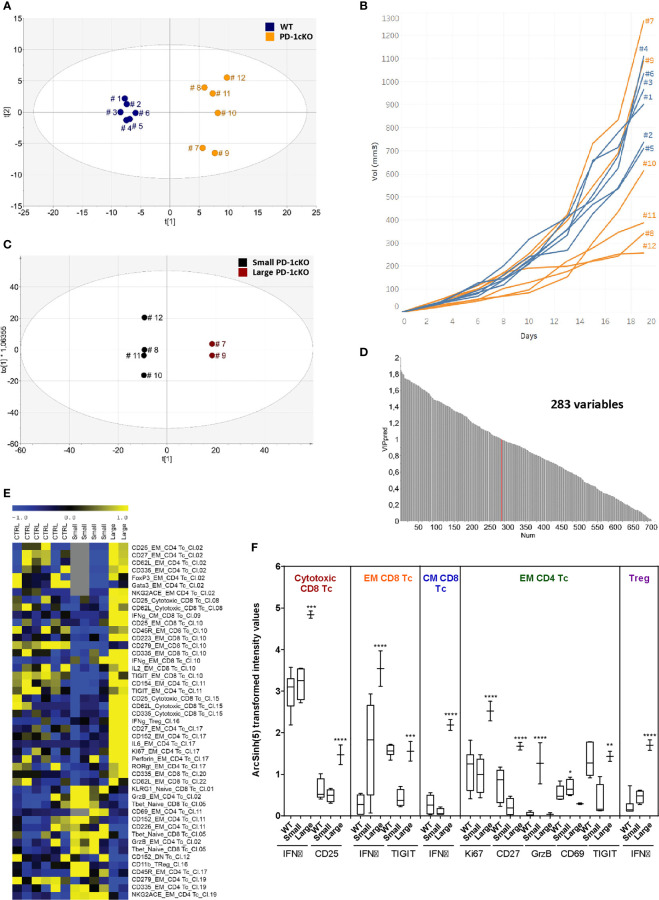
Large and small tumors have a distinct phenotypic signature in the PD-1cKO mice. **(A)** OPLS discriminant analysis (OPLS-DA) score scatter plot displaying the separation of PD-1cKO and WT mice based on X-variables. **(B)** MC-38 tumor growth in individual WT or PD-1cKO mice. **(C)** OPLS-DA score scatter plot displaying the separation of phenotype of tumor-infiltrating lymphocytes isolated from small and big tumors in PD-1cKO mice. **(D)** Statistical analysis selected variables significantly affected after PD-1 blockade compared to the respective control mice. **(E)** Heatmap depicting the signature of clusters upregulated in the small versus big tumors in the PD-1cKO mice. Data normalized to the mean and reduced to the SD for each variable. **(F)** Differential expression of selected markers in WT and small and big tumors in PD-1cKO mice expressed as asinh (5) transformed intensity value. *p < 0.05, **p < 0.01, ***p < 0.001, ****p < 0.0001. Data from one experiment with n = 6 mice/experiment, group, and time point.

Since deletion of *Pdcd1* in the PD-1cKO mice led to variable effects on tumor size, the relationship between the tumor size and phenotype of the TILs was investigated. OPLS-DA analysis was performed to analyze the grouping of WT and PD-1cKO mice based on the density of immune cell marker expression independent of tumor size. Indeed, a separation of the groups of WT and PD-1cKO mice could be seen indicating differences in density of immune cell marker expression depending on whether the cells had intact PD-1 signaling or not (**Figure 4A**). Furthermore, among the PD-1cKO, groups of mice also separated from each other on the y-axis (mouse 8, 10, 11, and 12 versus mouse 7 and 9). Thus, in the next step, the removal of the WT mice from the analysis resulted in the regrouping of PD-1cKO mice based on the tumor size. The PD-1cKO mice with large tumors, mouse 7 and 9 grouped together and far from the mice with small tumors (mouse 8, 10, 11, and 12) (**Figures 4B, C**). The variable importance in projection (VIP) defines which markers contributed to the regrouping of the PD-1cKO based on the tumor size (**Figure 4D**). This could give a prediction signature to investigate further in a larger group of mice. A cutoff of VIP >1 resulted in an inclusion of 283 variables for the discriminatory analysis. Depicted in the heat map is the analysis of markers expressed by the immune cells most variable in the 20 clusters (**Figure 3C**), correlated with the tumor size (**Figure 4E**).

In the large tumors of PD-1cKO mice, clusters within the cytotoxic, EM, and CM CD8 T cells, and regulatory T cells expressed higher levels of IFNγ than those from smaller tumors or WT mice (**Figure 4F**). The expression of CD25 and IFNγ was upregulated specifically in the cytotoxic CD8 and CM CD8 T-cell clusters of large tumors respectively compared to WT mice. Furthermore, a significant upregulation of Ki67 and CD27 was observed among the EM CD4 clusters found in the large tumors compared to the small tumors in PD-1cKO mice or WT mice. Finally, GrzB was specifically upregulated in small tumors among the EM CD4 T-cell clusters (**Figure 4F**). Altogether, the upregulated expression of Ki67, IFNγ, CD25, and CD27 among the CD8 and CD4 T-cell clusters indicate that the large tumors in the PD-1cKO are highly immunogenic with a potential for tumor regression, evident at later time points (**Figure 1C**).

### Gene Expression Pattern From the Tumor Tissue Reveals a Common Mechanism of Immune-Mediated Tumor Regression After Antibody Blockade or Genetic Deletion of PD-1 Signaling

**Figure 5:**
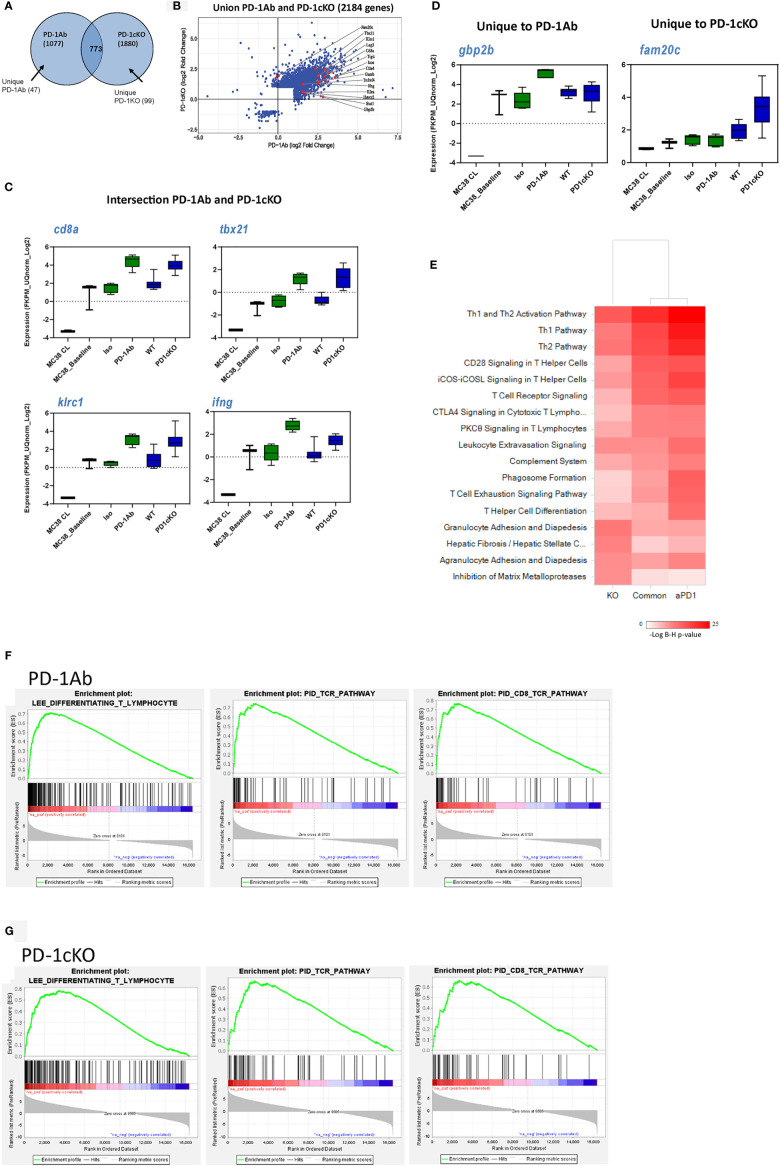
Gene expression pattern from the tumor tissue reveals a common mechanism of immune-mediated tumor regression after antibody blockade or genetic deletion of PD-1 signaling. Gene expression data after RNA sequencing of bulk tumor tissue excised from PD-1 Ab treated (15 days post-implant) or PD-1cKO mice treated with tamoxifen (28 days post-tumor implant). **(A)** Venn diagram illustrating the number of common (773) and unique genes regulated in PD-1-antibody treated (vs. isotype treated, 47) and PD-1cKO mice (vs. WT mice, 99). **(B)** Scatter plot showing the 2,184 genes regulated by either PD-1 Ab treated or in PD-1cKO mice, compared to respective controls, with a few genes highlighted. Most genes are upregulated in both comparisons. **(C)** Specific genes found to be commonly (*CD8a*, *Tbx21*, *Ifng*, *Klrc1*) or **(D)** uniquely (*Gbp2b*) upregulated in the PD-1 Ab-treated and PD-1cKO mice (*Fam20c*) compared to respective controls. No statistical significance FDR <0.001 between PD-1 Ab treated compared to PD-1cKO, except for *Gbp2* (only PD1 antibody treatment) and *Fam20c* (only PD-1cKO). **(E)** Ingenuity pathway analysis^®^ of gene sets of PD-1 Ab-treated and PD-1cKO mice, compared to respective controls, and the intersection of the two (common), showing similarly enriched pathways. **(F, G)** GSEA analysis showing the top enriched gene sets following either PD-1 Ab treatment or in PD-1cKO mice, compared to respective controls. See also **Supplementary Figure S3**.

To identify genes associated with tumor regression after PD-1 blockade in the tumor tissue, we performed RNAseq analysis prior to tumor regression in PD-1 Ab and PD-1cKO mice. Overall, there were more differentially expressed genes in the PD-1cKO, compared to WT mice, than in the PD-1 Ab-treated mice, compared to isotype controls (**Table 1** and **Figure 5A**). Differential gene expression analysis identified 2,184 genes significantly changed [at least twofold change and corrected p-values with false discovery rate (FDR) <0.01, and after excluding low expressed genes] by either PD-1Ab or in PD-1cKO mice compared to their respective controls (**Table 1**, **Figure 5B** and **Supplementary Figure S3A**). Of these, 773 were differentially regulated in both the PD-1cKO and PD-1 Ab-treated mice (**Supplementary Figure S3B**), with only 99 and 47 genes differentially regulated only in the former and latter, respectively (**Table 1**). Most of the differentially expressed genes were upregulated compared to those in controls (**Figure 5B**) and in the PD-1 Ab-treated mice, which was apparent after the second dose (**Table 1** and **Supplementary Figure S3**). The level of high gene expression was related to small tumor size in individual mice.

**Table 1:** RNAseq analysis of tumor tissue.

Treatment	Baseline	Days post-implant	1×	1.5×	2×
PD-1Ab	Isotype	11	13	13	12
		15	1408	1329	1077
PD-1cKO	WT	28	5771	3734	1880
		Union	6,080	4,096	2,184
		Intersection	1,099	987	773
		PD-1Ab_15d_unique	119	95	47
		PD-1cKO_28d_unique	2,005	724	99

Furthermore, the injection of tumor cells to mice resulted in the upregulation of some genes compared to cells in culture but similar to isotype control mice (**Figure 5C** and **Supplementary Figure S3**). In tumors from both the PD-1 Ab-treated and PD-1cKO mice, genes related to cell lineage and activation of the immune system like *CD8*, *Tbx21*, *Klrc1*, and *Ifng* were strongly upregulated compared to tumors from mice treated with isotype control Ab (**Figure 5C**). Upregulation of *Gbp2b* (interferon-induced guanylate-binding protein) was unique to the tumor from PD-1 Ab-treated mice. GBP2 is a gene related to the superfamily of large GTPases induced mainly by IFNγ. *Fam20* (a kinase that catalyze the phosphorylation of secreted proteins) was upregulated exclusively in the tumor tissue from PD-1cKO mice (**Figure 5D**).

Differentially expressed genes in the PD-1 Ab-treated mice, PD-1cKO mice, or common to the two models were analyzed using the Ingenuity Pathway Analysis^®^ software. Pathway analysis identified enrichment in T-helper 1 and 2 type immune responses, T-cell activation, and signaling pathways centric to PD-1 blockade (PD-1 Ab-treated and PD-1cKO mice) (**Figure 5E**). Next, the biological processes associated with the gene signature in the PD-1 Ab-treated and PD-1cKO mice was characterized using gene set enrichment analysis, using the c2_all, c2cp, and hallmark gene sets (GSEA; **Figures 5F, G**). The analysis revealed enrichment of genes associated with T-cell differentiation, TcR CD8 signaling pathway, hallmark inflammatory response, and natural killer cell mediated cytotoxicity, among others. Remarkably, several of the enriched pathways were common between the PD-1 Ab-treated and PD-1cKO mice. Altogether, the data suggest that T-cell differentiation, increased intracellular signaling in T cells, and upregulation of inflammatory pathways are common after PD-1 Ab treatment or in PD-1cKO mice, rendering the tumors sensitive to PD-1 blockade.

## Discussion

Treatment with antibodies to PD-1/PD-L1 has shown activity in more than 20 tumor types since the first approval in 2014. However, only a subset of patients will respond to treatment with antibodies to PD-1/PD-L1. Escape from tumor immunity after PD-1 blockade can occur due to (1) inherent low immunogenicity of the tumor, (2) impaired T-cell trafficking and retention of T cells in the tumor, or (3) inhibitory factors expressed in the tumor microenvironment that prevent T-cell activation. Understanding immune mechanisms of resistance to PD-1 blockade is essential in developing strategies for targeted treatment of patients with progressive disease. To this end, we have characterized the tumor growth, immune cell infiltration, and molecular events after blockade of PD-1 signaling in PD-1 Ab-treated, PD-1KO, and PD-1cKO mice. Interference with PD-1 signaling was highly efficacious in the MC-38 tumor model leading to TGI and CR, systemic immune responses, and antigen-specific memory. Mass cytometry analysis identified 20 separate T cell subsets, with proliferating activated CD8 T cells expressing IFNγ and GrzB to be expanded prior to tumor growth regression, indicating that a dominant and functional CD8 response is important for tumor immunity after PD-1 blockade. Tumor RNA sequencing analysis confirmed pathways of inflammation and CD4 and CD8 T-cell activation to be common between the PD-1 ab-treated and PD-1cKO mice and a dominant IFNγ response in the tumor microenvironment. The study emphasizes clearly a role for activated effector memory CD4 and CD8 T cells for tumor immunity during conditions of blockade of PD-1 signaling.

MC-38 tumors were responsive to PD-1 blockade with tumor growth regression in all but 10%–20% of the mice in all three models. Deletion of *Pdcd1* in PD-1cKO adult mice led to tumor growth regression and complete responses, comparable to PD-1Ab treatment or PD-1KO mice, except for tumor growth inhibition of large tumors (>1,000 mm^3^). When tumor size exceeded 1,000 mm^3^ in the PD-1KO mice and after PD-1 Ab treatment, mice proceeded to lose tumor control and were sacrificed as the tumor size exceeded the ethical limit. Remarkably, PD-1cKO mice could control tumors as large as 1,500 mm^3^, and after initial tumor growth, inhibition or complete responses were observed correlating with the progressive deletion of PD-1 expression on CD4 and CD8 T cells. Indeed, mass cytometry analysis revealed that the large tumors in the PD-1cKO were highly immunogenic with a potential for tumor regression (18–21 days post-tumor implant). IFNγ was significantly upregulated in EM, cytotoxic, CM CD8 T cells, and Tregs as was CD25 expression on cytotoxic CD8 T cells, whereas Granzyme B was increased in EM CD4 T cells. These changes are consistent with an ongoing early response in the large tumors versus a more terminal response with mature cytotoxic T cells in the small tumors. Thus, the PD-1cKO mice, which enables deletion of *Pdcd1* following tumor implantation and has normal baseline frequencies of EM CD4 and CD8 T cells, can be a useful model to investigate both tumor cell-intrinsic and tumor cell-extrinsic factors leading to regression of large tumors.

The complete response after PD-1 blockade in some of the PD-1 Ab-treated, PD-1KO, and PD-1cKO mice led us to investigate whether the primary response had generated a memory immune response. All mice with complete regression of primary tumor rejected a second challenge with the same tumor cells but not heterologous tumor cells, indicating that the generated memory response was antigen specific as previously reported in the context of cancer vaccines or in preclinical combination therapies (24). The tumor-specific immune response after PD-1 blockade can be either due to local proliferation of TILs or active recruitment from the draining lymph nodes or both. Cytotoxic CD8 T-cell populations expressed Ki67, indicating that active proliferating cells are present in the tumor. In addition, EM CD8^+^ T cells were expanding in the tumor-draining lymph nodes after challenge in the WT and after rechallenge in PD-1cKO mice. EM CD8^+^ T cells were reduced in the LN of challenged PD-1cKO mice over challenged WT mice, indicating that these cells may have migrated to the tumor already at that time point, demonstrating the dynamics of the immune response. The recruitment of CD8^+^ T cells from the secondary lymphoid organs to the tumor was previously shown to contribute to tumor growth inhibition after blockade with PD-1 Abs since treatment with FTY720 lowered the survival, compared to mice treated with PD-1 Ab alone (9). Indeed, in patients with basal and squamous cell carcinoma treated with PD-1 blockade, the T-cell response was shown to be derived from a specific repertoire of T-cell clones that enter the tumor, indicating that recruitment of T cells from the draining lymph nodes contributes to the response after PD-1 blockade (25).

Phenotypic characteristics and the effector functions of the tumor-infiltrating lymphocytes necessary for tumor growth inhibition can be effectively deduced by flow and mass cytometry by analysis of cell surface and intracellular markers. Based on lineage and activation markers, transcription factor expression, and functional response (cytokine secretion and granzyme B expression), we were able to define 20 different clusters of immune cells subsets in the tumor, which could be categorized into eight separate CD4 and CD8 T-cell populations. PD-1 blockade led to an expansion of ICOS-positive CD8^+^ T cells and particularly a non-proliferating effector memory CD8 T cell subset and a proliferating cytotoxic CD8 T cell subset, expressing both IFNγ and granzyme B. The effect of PD-1 blockade on CD4^+^ T cell subsets in our study was less dramatic, although gene expression analysis revealed Th1 pathways to be highly upregulated. A subtle yet consistent age-dependent expansion of CD8 T-cell phenotypes in the PD-1KO mice has been previously reported (3, 4). In addition, PD-1 Ab treatment has been shown to lead to the expansion of a CD8 T-cell cluster in the tumor, expressing Tbet+Eomes+MHCII+BATF+PD-1+TIM-3+CD27+CD69+Gata-3+ that correlated with small tumor size (26). However, since effector molecules such as cytokines and granzyme B were not included in the analysis, a potential functional classification to the immune cell subsets could not be assigned. The analysis of effector memory cytotoxic T cells (Granzyme B, NKG2ACE, CD223, CD366, TIM-3, CD27, CD44, CD39, and expressing IFNγ), found in the tumors of PD-1Ab, PD-1KO, and PD-1cKO mice in our study, prior to tumor growth inhibition, contributes further to the knowledge of functional TILs that can contribute to long-term tumor immunity after PD-1 blockade.

In addition to changes in CD8 subsets, the ratio of CD8:Tregs was increased, and the frequency of Foxp3^+^ TILs was decreased in the PD-1KO and PD-1cKO mice. Although the mechanisms leading to the reduction in the frequency of CD4^+^Foxp3^+^ T cells is unknown, it could be attributed to a lower conversion rate of CD4^+^ effector cells to Foxp3^+^Tregs in the absence of PD-1 (24). Contrary to our findings, a recent study proposed that PD-1 contributes not only to immune suppression of effector T cells but also to PD-1 expressing regulatory T cells in the tumor of non-small cell lung cancer patients. A strong inhibition of CD8 T-cell activation was observed in cocultures of PD-1^high^Tregs with PD-1^low^ CD8 responder cells after treatment with monoclonal antibody to PD-1, supporting the observation that PD-1 blockade enhances the suppressive capacity of PD-1^high^Treg (27). However, the increase in the CD8^+^:Foxp3^+^ ratio in the tumor of PD-1Ab, PD-1KO, and PD-cKO mice suggests that the proposed enhanced immunosuppressive capacity by Foxp3^+^Tregs in the absence of PD-1 signaling could be counteracted by the adequate expansion of functional effector CD8 T cells leading to tumor growth inhibition. Further evidence for the activation of CD8 T cells in the PD-1 Ab, PD-1KO, and PD-1cKO mice was provided by the increase in the frequency of CD8^+^ICOS-expressing cells. The ICOS-ICOSL-signaling pathway was significantly upregulated in the Ingenuity Pathway Analyses in both cKO and PD1 mAb treatment. In future studies, the phenotypic and functional analyses of CD8 T cells expressing ICOS by mass cytometry will help to further define the role of ICOS+CD8 T cells for tumor immunity. Indeed, ICOS on T cells has been shown to favor the survival of effector-memory T cells (28, 29) promoting tumor immunity, and therefore, agonistic Abs targeting ICOS are under investigation in clinical trials for cancer immunotherapy, in combination with PD-1 blockade. Although the frequency of Foxp3+ cells in the tumor is decreased in the PD-1 Ab, PD-1KO, and PD-1cKO mice, the suppressive function Foxp3^+^Tregs might still be enhanced. However, the concomitant activation of CD8 T cells early on during the response can overcome the suppressive effects of Treg to promote tumor immunity.

The immune response both at the level of single cells and in the bulk tumor was found to be dominated by a proinflammatory response as evidenced by an increase expression of *Tbx21* (T-bet) and *Ifng* (IFNγ) and the detection of IFNγ expression by CD8 effector memory and cytotoxic T cells in the tumors of PD-1cKO or PD-1 Ab-treated mice. Gene expression analysis revealed that >2,000 genes were commonly upregulated between the tumors from PD-1 Ab-treated and PD-1cKO mice. Indeed, many of the pathways that contribute to tumor growth inhibition were shared between the PD-1 Ab-treated and PD-1cKO mice. One pathway that was found to be significantly upregulated in the tumor of both PD-1Ab-treated and PD-1cKO mice was the IFNγ signaling pathway and its associated downstream molecules such as *stat-1* and *tbx21*. In this respect, downregulation of specifically *Ptpn2* was shown to correlate with tumor growth inhibition through enhancement IFNγ signaling by phosphorylation of STAT1, indicating a specific role for *ptpn2* in regulating IFNγ signaling and effective tumor immunity (10). The observed significant downregulation of *Ptpn2* expression in the tumors of PD-1cKO mice in our study, with a concomitant significant increase in *stat1* expression indicates that the regulation of *Ptpn2* expression could promote tumor regression through IFNγ in the model as well. The role of IFNγ signaling in tumor immunity after PD-1 blockade is also evident from the six-gene signature found to predict clinical response in patients, which includes *ifng* and *stat1* (30). In addition, a significant upregulation of genes *Fam 20* (an extracellular protein kinase) was detected uniquely in the tumors from PD-1cKO mice and *Gbp2b* (interferon-induced guanylate-binding protein 1) in the tumors from PD-1 Ab-treated mice. The relevance for the upregulation of *Fam20c* in the tumor of PD-1cKO mice is unclear and may be related to the phosphorylation of cytokines such IL-6, a known a substrate of Fam20c (31). IFNγ is a known inducer of guanylate binding proteins (Gbps) and studied extensively in the context of intracellular infections, due to the role of Gbps in the activation of the inflammasome pathway, leading to secretion of IL-1β and IL-18. In cancer, the Gbp family members are among the IFNγ-dependent genes most highly induced in cancer patients, and increased tissue expression of human GBP2 in several cancer types including colorectal cancer is correlated with favorable prognostic outcomes (32). It is also possible that the union of PD-1 Ab- and PD-1cKO-induced genes has a more significant intersection when considering temporal variation between the two models. Although samples were collected at physiological equivalent stages, they may not be exactly the same. Either way, a strong correlation between PD-1 Ab-treated and PD-1cKO mice was found in gene expression induction and enrichment profiles and immune pathways. Ingenuity Pathway Analyses and Gene Set Enrichment Analyses further indicated that, besides T-cell activation pathways and signatures, other processes may be involved in the antitumor response or be influenced by it. These include PKC ϕ signaling in T lymphocytes, complement system, phagosome formation, and granulocyte activation pathways. Exploration of these pathways will lead to novel insights as well into the antitumor effects of PD-1 blockade.

In conclusion, high-dimensional cellular and genetic profiling approaches of intratumoral immune cell subsets can identify potential mechanisms of immune intervention after PD-1 blockade. The study provides insights into the cellular and functional changes that occur within the major intratumoral T-cell subpopulations following PD-1 antibody treatment or genetic deletion of *Pdcd1*. Our data indicate that the cell-intrinsic action of PD-1 on several different functional CD8 T cell subsets plays a crucial role in its antitumor efficacy.

## Data Availability Statement

The RNA sequencing datasets for this study can be found at Gene Expression Omnibus https://www.ncbi.nlm.nih.gov/geo/ accession number GSE171274.

## Ethics Statement

All animal procedures were approved by the Institutional Animal Care and Use Committee (IACUC) of Merck research laboratories (APS# 400265), in accordance with guidelines of the Association for Assessment and Accreditation of Laboratory Animal Care.

## Author Contributions

Conceptualization: SR, TR, HL, and RW. Methodology: NT-S, CK, AS, AC, MS, WB, EM, and TR. Investigation: SR, MS, WB, EM, EP, HL, and RW. Software: EM and HL. Funding acquisition: SR, AC, TR, EP, HL, and RW. Supervision: WB, AC, TR, EP, HL, and RW. Writing—original draft: SR, EM, WB, TR, HL, and RW. Writing—review and editing: SR, EM, WB, EP, AC, TR, HL, and RW. All authors contributed to the article and approved the submitted version.

## Funding

This work was supported by grants to SR from the Sahlgrenska Academy at the University of Gothenburg, Sweden, and The Swedish Foundation for Strategic Research (SSF) *via* the mobility grants program and the Swedish Cancer Society (Cancerfonden).

## Conflict of Interest

Authors SR, NT-S, AS, MS, EP and AC were employed by Merck & Co., Inc., Kenilworth, NJ, USA. CK and RW were employed by Merck & Co., Inc., Kenilworth, NJ, USA at the time of the study and are currently employed by Synthekine. WB, EM, and TR are currently employed by Merck & Co., Inc., Kenilworth, NJ, USA.

## Publisher’s Note

All claims expressed in this article are solely those of the authors and do not necessarily represent those of their affiliated organizations, or those of the publisher, the editors and the reviewers. Any product that may be evaluated in this article, or claim that may be made by its manufacturer, is not guaranteed or endorsed by the publisher.
