# Supplementary material for: Conditional Deletion of Pdcd1 Identifies the Cell-Intrinsic Action of PD-1 on Functional CD8 T Cell Subsets for Antitumor Efficacy
Source: Front Immunol. 2021 Nov 29;12:752348. doi: 10.3389/fimmu.2021.752348 (PMC8667167; doi:10.3389/fimmu.2021.752348)
Supplement: Supplementary file 1 [file DataSheet_1.docx]

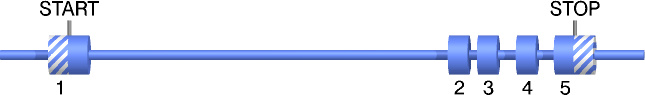

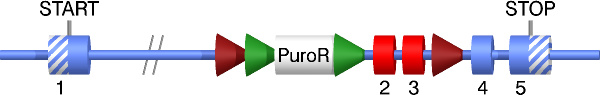

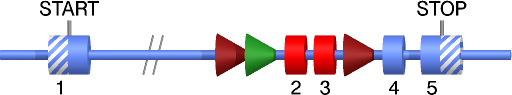

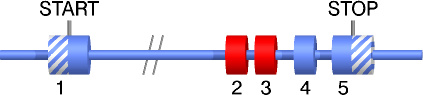

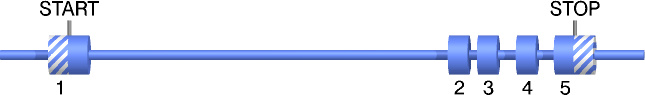

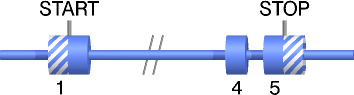


**D**

**C**

**B**

**A**

**Supplementary Figure 1**

**1.**

**2.**

**3.**

WT mouse genomic locus

Targeted allele (homologous recombination)

Conditional KO allele (Flp recombination)

Proximal sgRNA

Distal sgRNA

**1.**

**2.**

**3.**


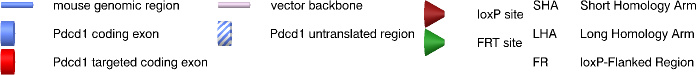

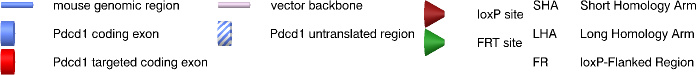

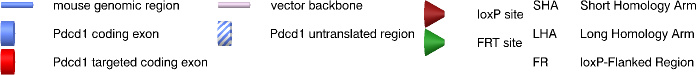


**PD-1**

KO day 11

WT day 22

PD-1cKO day 22

PD-1cKO day 15

PD-1cKO day 11

**CD4**

**CD8**

**
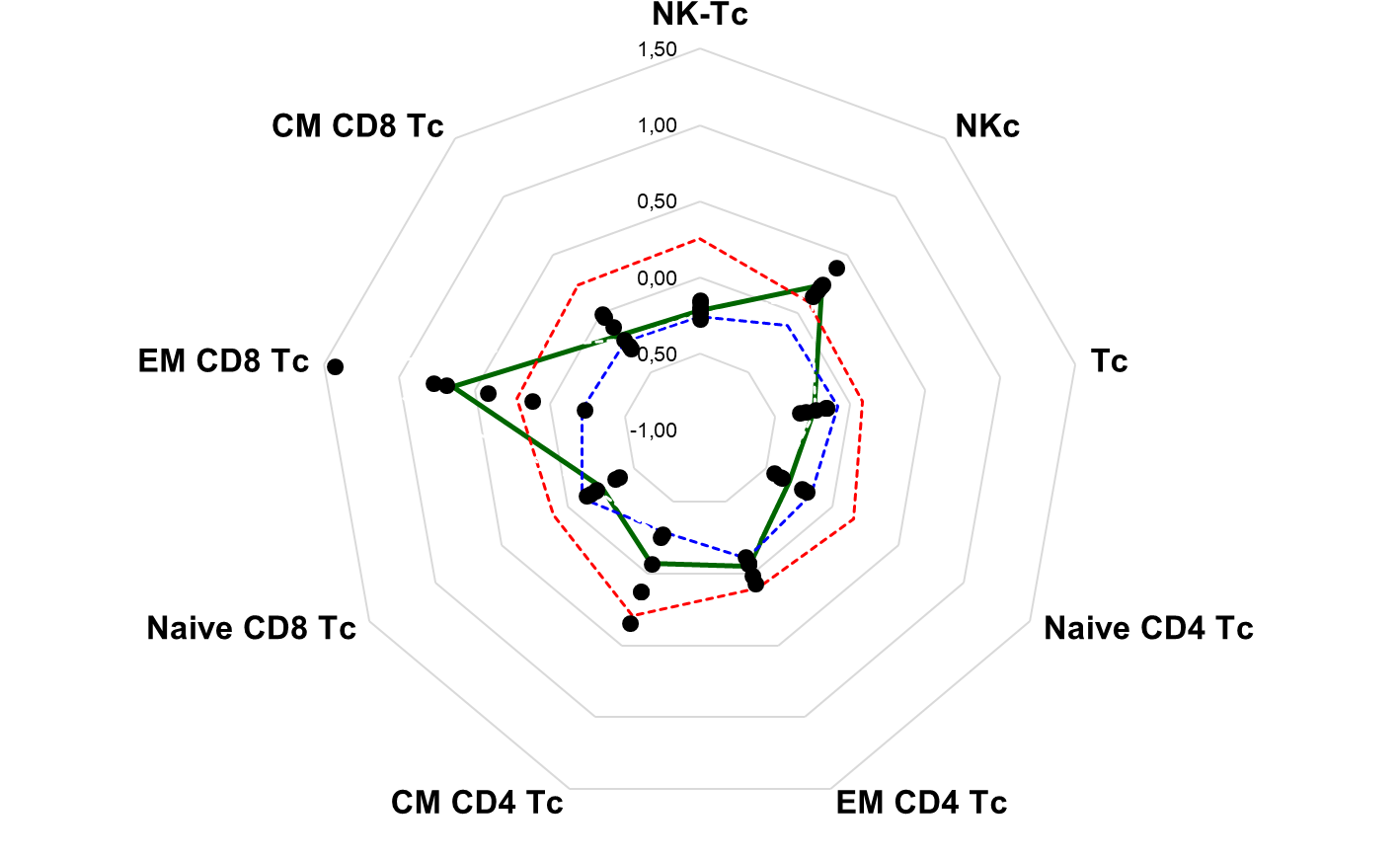

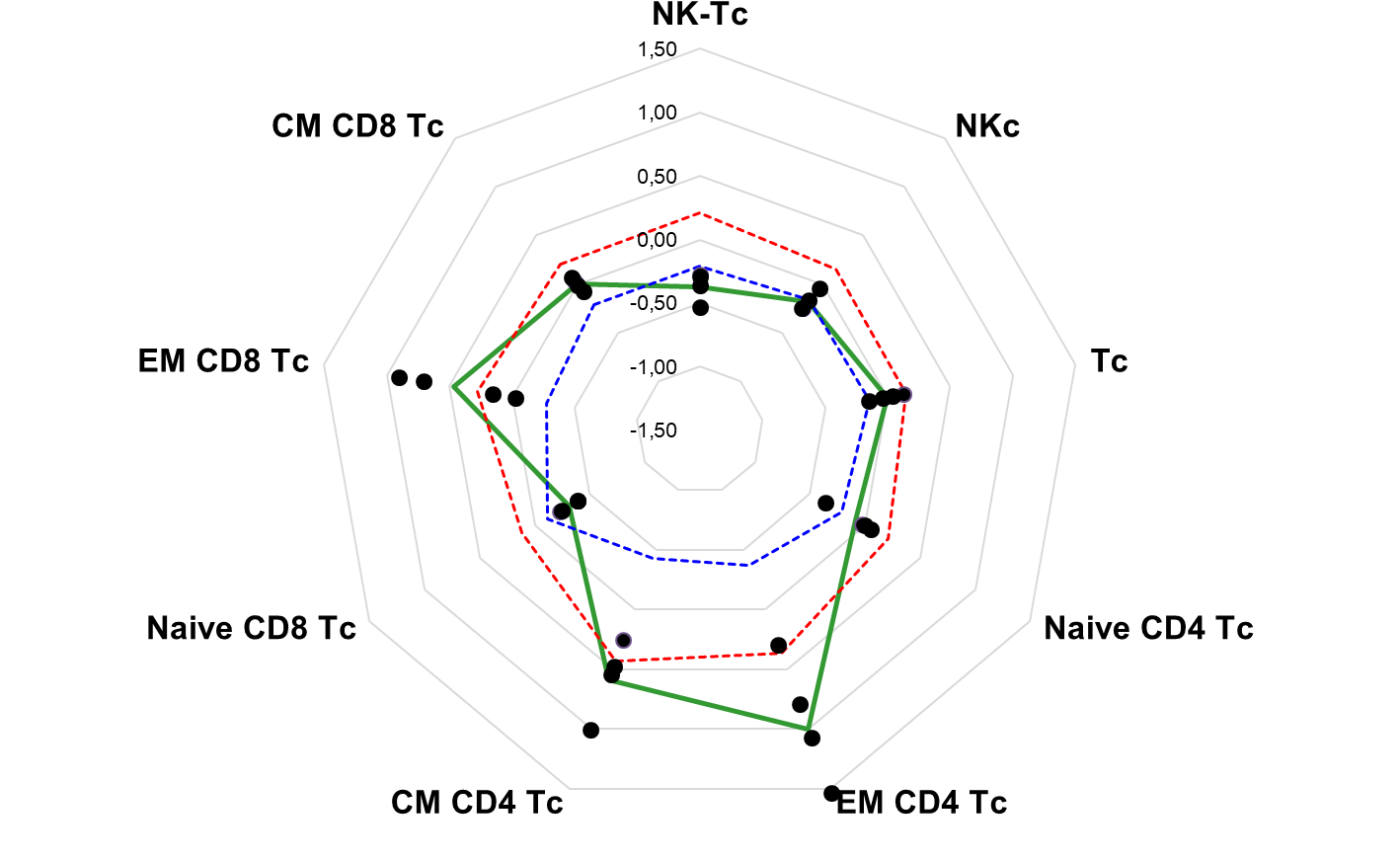

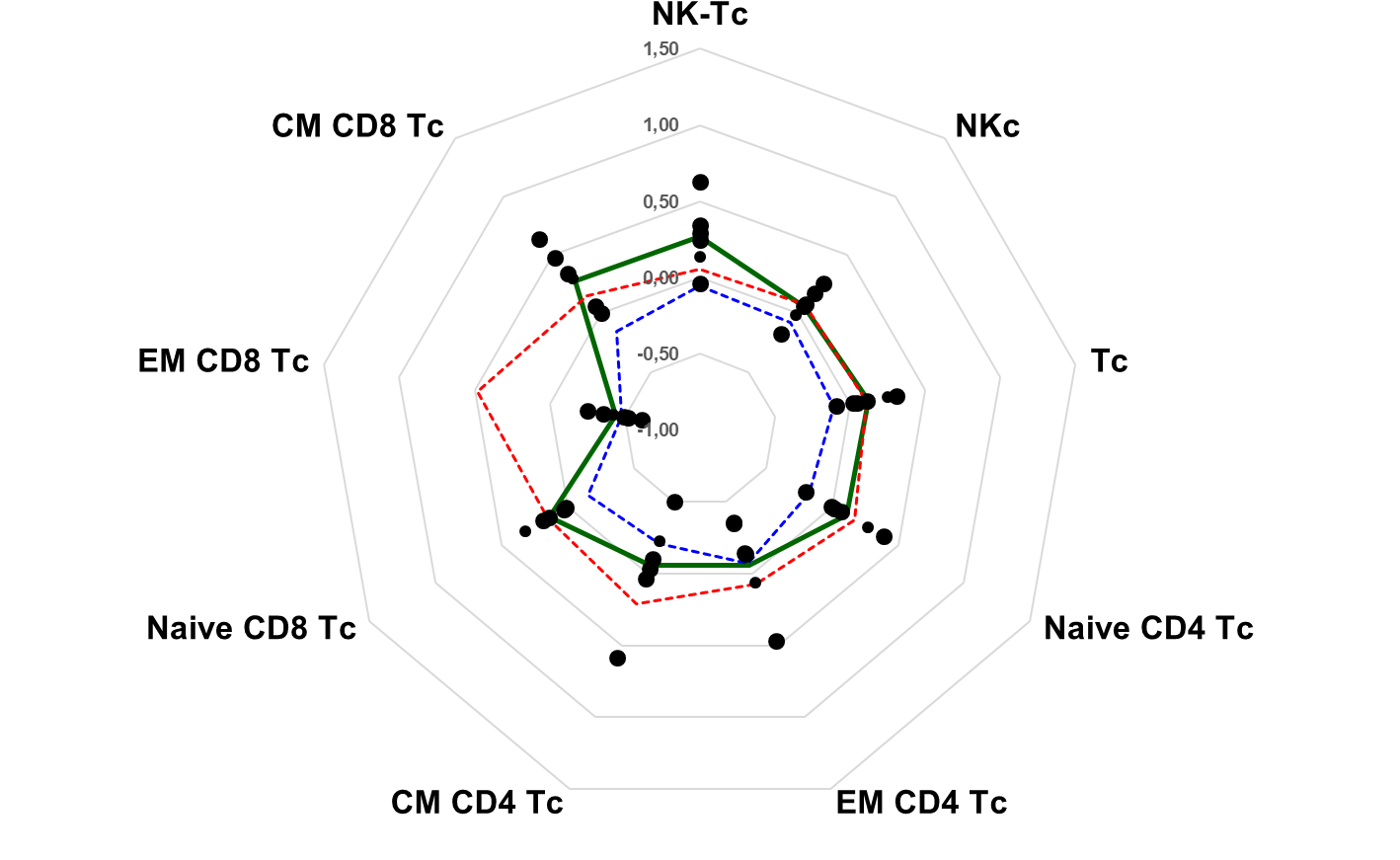
**

**E**

**G**

Blue line: (avg value of reference)-1SD

Green line: (avg value of condition 1/avg value of reference) -1SD

Red line: (avg value of reference)+1SD

Chal. WT/naïve WT

Chal.PD-1cKO/Chal.WT

Rechall. PD-1cKO/Chal. KO

**F**

**Supplementary Figure 1 (related to figure 1). Targeting strategy of the PD-1KO and PD-1cKO mice and phenotypic characterization of infiltrating lymphocytes in the tumor and draining lymph nodes.** **A** 1: mouse genomic locus of the *Pdcd1* gene. 2: Location of proximal and distal sgRNA and loxP sites, respectively, which were co-injected with Cas9 mRNA into C57BL6/Ntac zygotes. 3: *Pdcd1* KO allele after CRISPR/Cas9 mediated deletion of exons 2 and 3. Pdcd1 coding exons 1-4 and translational start and stop codons are depicted. **B** 1: Mouse genomic locus of the *Pdcd1* gene. 2: Conditional KO allele after Flp recombinase mediated removal of the positive selection marker. 3: Targeted allele after homologous recombination in ES cells. **C.** Representative histogram plots of PD-1 staining of tumor infiltrating lymphocytes isolated from WT (day 22), PD-1KO (day 11) and PD-1cKO (day 22, 15 and 11) post-tumor implant **D.** PD-1 expression on tumor infiltrating CD4^+^ and CD8^+^ T cells isolated from littermate controls or PD-1cKO mice day 11, 15, and 22 post-tumor implant . **E.** Complete responses to rechallenge of MC-38 cells injected to cured PD-1 Ab treated mice, PD-1KO and PD-1cKO mice (4-8 weeks after complete response). MC-38 challenge in naïve control mice and B16F10 challenge in cured PD-1 Ab treated mice, resulted in exponential tumor growth. Data mean+SEM and representative of 2 independent experiments. **F.** Number of cells in the draining LN of mice after primary (day 13) or secondary (day 3) challenge with MC-38 cells in tamoxifen treated PD-1cKO and WT mice. **G.** Flow cytometry analysis of T cell and NK cells subsets in draining lymph nodes of mice after primary (day 13) or secondary (day 3) challenge with MC-38 cells in tamoxifen treated PD-1cKO and WT mice. Blue line: CTRLmin: Mean CTRL – 1 SD, Red line: CTRLmax: Mean CTRL + 1 SD, Green line: Fold Change of Mean PD-1cKO/Mean CTRL, each dot represents an independent PD-1cKO mouse (nPD-1cKO/Mean CTRL).

**Supplementary figure 2**


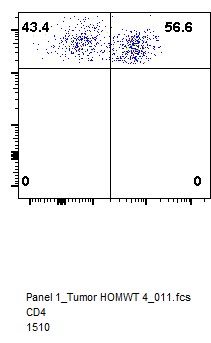

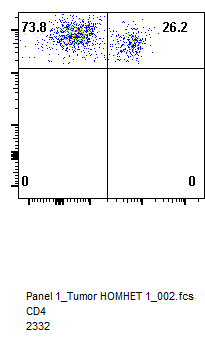

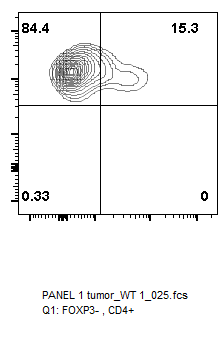

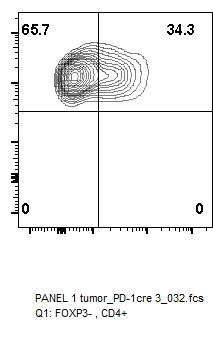


**WT**

**A**


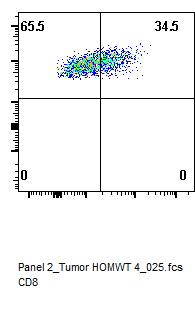

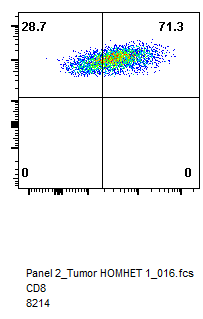


**ICOS**

**PD-1cKO**

**Foxp3**

**WT**

**PD-1cKO**

**Gated CD4+Foxp3-**


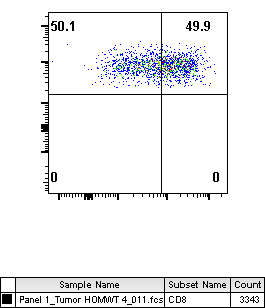

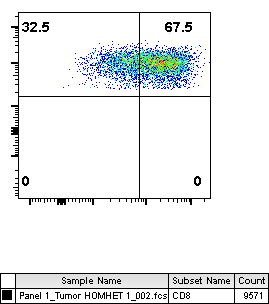


**CD4**

**CD8**

**Ki67**

**CD4**

**CTLA-4**

**B**

**DAY 22**

**DAY 15**

**DAY 11**

**C**

CD4+Foxp3+

CD4+Foxp3-

CD4+

**D**

**DAY 22**

**DAY 11**

**DAY 15**

CD8+

CD8+

**Supplementary Figure 2 (related to figure 2). Time course analysis of T cell infiltration after deletion of *Pdcd1* in adult PD-1cKO mice. A.** Representative flow cytometry staining of TILs isolated from PD-1cKO mice day 22 post implant. **B.** Time course analysis of the ratio of CD8:CD4 among the tumor infiltrating T cells, day 11, 15 and 22 post-tumor implant. C**.** Expression of markers of checkpoint inhibition or activation among the CD4^+^ Foxp3^+^ or CD4^+^Foxp3^-^ TILs. D**.** Expression of markers of checkpoint inhibition or activation among the CD8^+^ TILs. Data representative of 3 independent experiments with n=4-5 mice/experiment, group and time point.


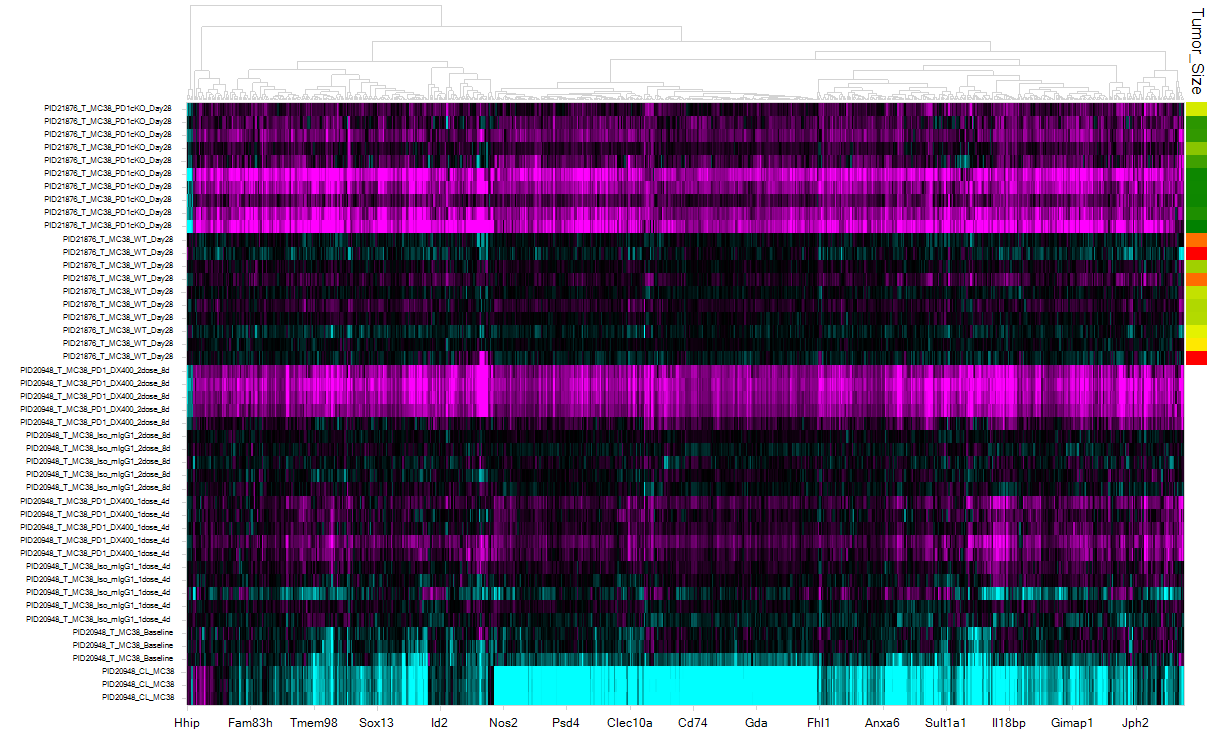

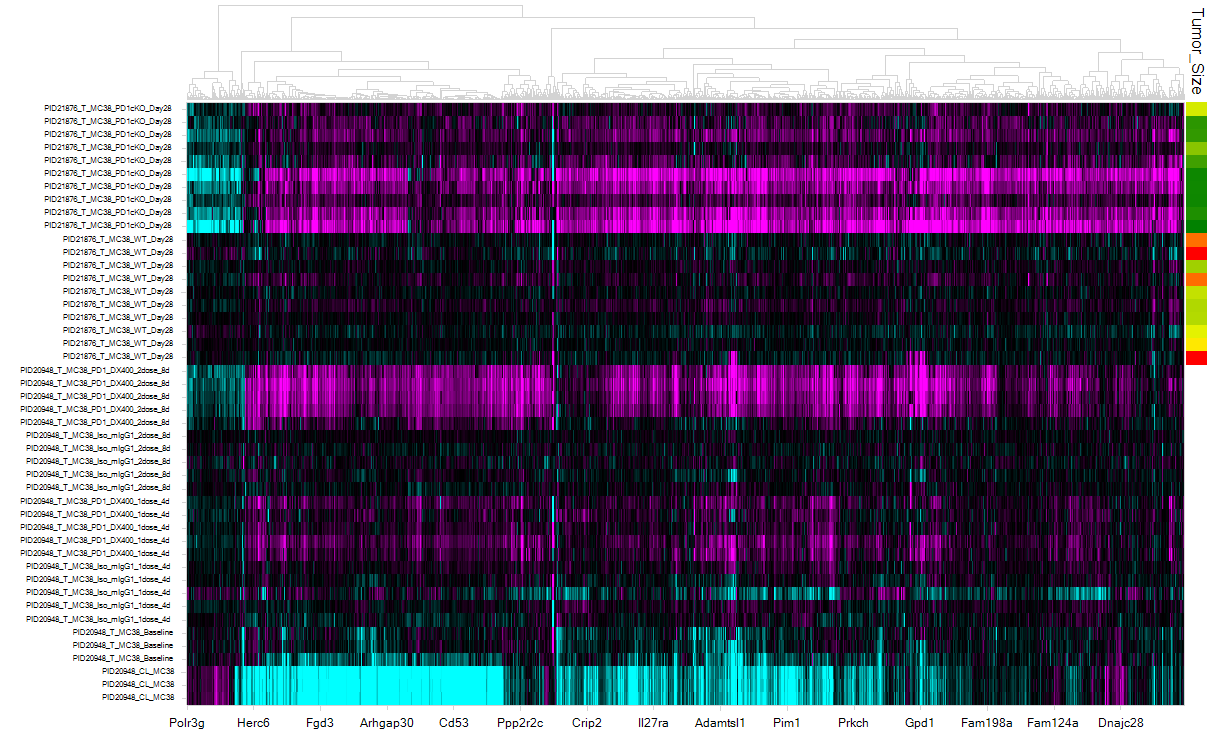


Log2 Fold Change

**Intersection: PD-1cKO and PD-1Ab = 773 genes**

Cell Line:Iso (day 15)

Baseline:Iso (day 15)

Iso:Iso (day 11)

PD-1Ab:Iso (day 11)

Iso:Iso (day 15)

PD-1Ab:Iso (day 15)

WT:WT (day 22)

PD-1cKO:WT (day 22)

**Supplementary figure 3**

Log2 Fold Change

**Union: PD-1cKO and PD-1Ab = 2184 genes**

Cell Line:Iso (day 15)

Baseline:Iso (day 15)

Iso:Iso (day 11)

PD-1Ab:Iso (day 11)

Iso:Iso (day 15)

PD-1Ab:Iso (day 15)

WT:WT (day 22)

PD-1cKO:WT (day 22)

**A**

**B**


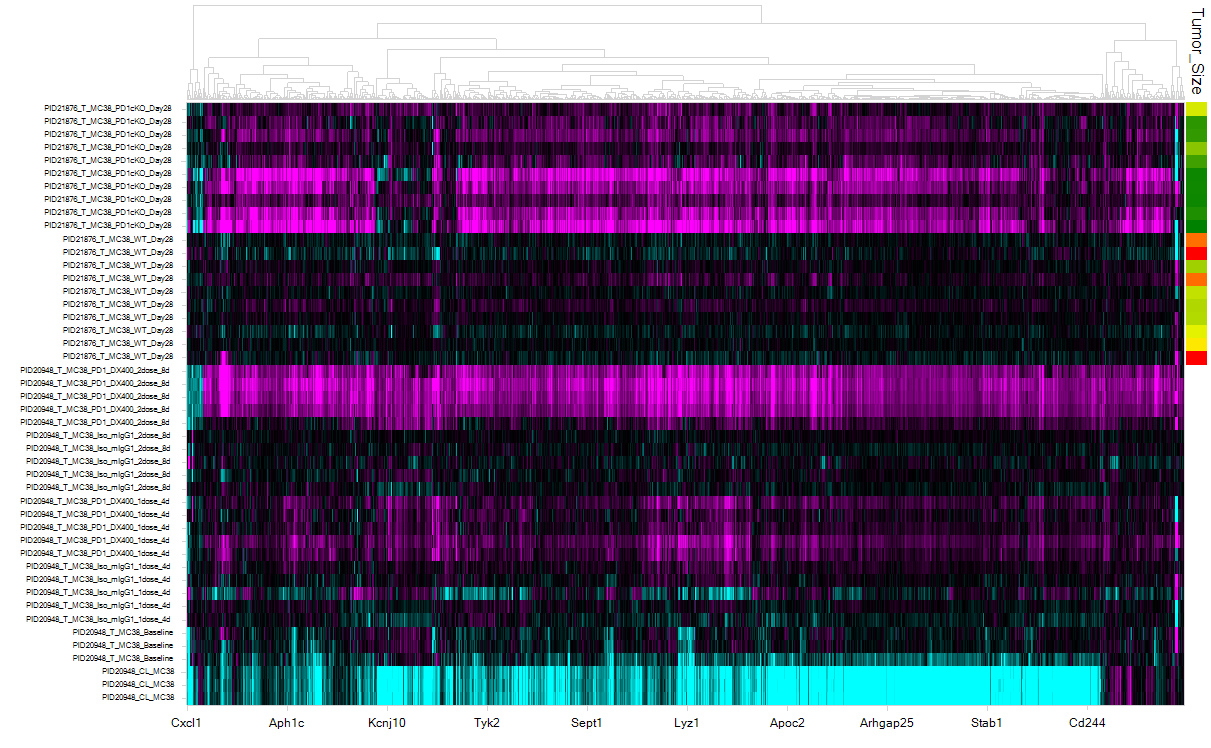


**C**


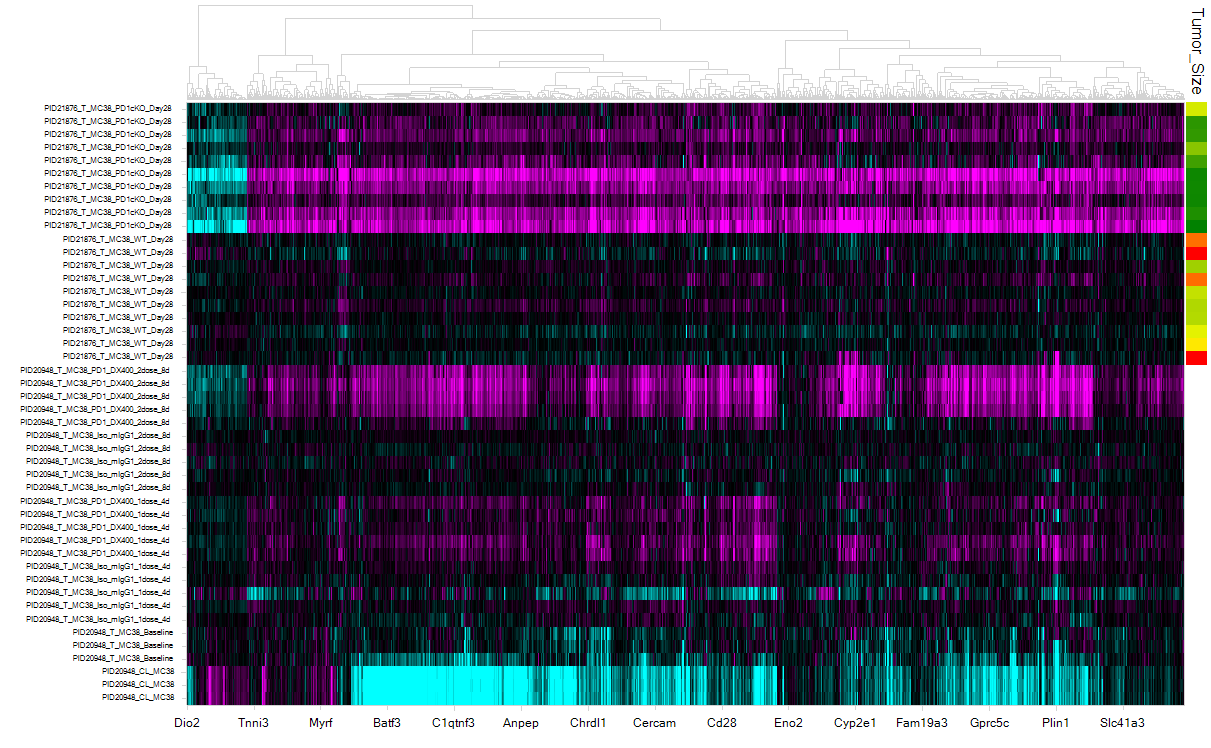


PD-1cKO:WT (day 28)

WT:WT (day 28)

PD-1Ab:Iso (day 15)

Iso:Iso (day 15)

PD-1Ab:Iso (day 11)

Iso:Iso (day 11)

Baseline:Iso (day 15)

Cell Line:Iso (day 15)

**PD-1Ab (day15) = 1077 genes**

Log2 Fold Change

PD-1cKO:WT (day 28)

WT:WT (day 28)

PD-1Ab:Iso (day 15)

Iso:Iso (day 15)

PD-1Ab:Iso (day 11)

Iso:Iso (day 11)

Baseline:Iso (day 15)

Cell Line:Iso (day 15)

Log2 Fold Change

**D**

**PD-1cKO (day 28) = 1880 genes**

**Supplementary Figure 3 (related to figure 5): Gene expression pattern from the tumor tissue reveals a common mechanism of immune mediated tumor regression after antibody blockade or genetic deletion of PD-1 signaling.**Gene expression data after RNA sequencing of bulk tumor tissue excised from PD-1 Ab treated (11 or 15 days post- tumor implant) or PD-1cKO mice treated with tamoxifen (22 days post-tumor implant). Shown in the heat maps are the genes that were significantly regulated by PD-1 Ab treatment compared to isotype treatment at 11 and 15 days post-tumor implant or PD-1cKO mice treated with tamoxifen compared to littermate control mice treated with tamoxifen (28 days post-tumor implant) (**A,** union 2184), (**B**, intersection 773), (**C**, PD-1 Ab treatment alone 1077), or (**D**, PD-1cKO alone 1880). Isotype control and cell line control was included in the analysis. A significance threshold of a 2-fold change, FDR_BH<0.01, and normalized counts >20 was set in at least one comparison group.  The color gradient represents fold change of each individual animal (each row) compared with the corresponding pooled control animals (−3 to 3 fold).

**SUPPLEMENTARY TABLE 1:** Antibody clones and vendor for flow and mass cytometry analysis.

**Flow cytometry**

| **Antibodies and clone** | **Vendor** |
| --- | --- |
| CD45 (clone 30-F11) | Biolegend |
| CD11b (clone M1/70) | Biolegend |
| TcRb (clone H57-597) | BD Biosciences |
| CD3 (clone 145-2C11) | BD Biosciences |
| CD11c (clone N418 | Biolegend |
| F4/80 (clone BM8) | Biolegend |
| Ly6C (clone HK1.4) | Biolegend |
| Ly6G (clone 1A8) | Biolegend |
| PD-L1 (clone 10F.9G2) | Biolegend |
| I-A/I-E (clone M5/114.15.2) | Biolegend |
| CD4 (clone RM4-5) | Biolegend |
| CD8a (clone 53-5.8) | Biolegend |
| CD25 (clone PC61) | Biolegend |
| CD69 (clone H1.2F3) | Biolegend |
| CD44 (clone IM7) | Biolegend |
| CD62L (clone MEL-14) | Biolegend |
| PD-1 (clone RMP1-30) | Biolegend |
| CD278 (clone C398.4A) | BD Biosciences |
| Foxp3 (clone FJK-16s) | eBioscience |
| Ki67 (clone B56) | BD Biosciences |
| GZMb (clone GB11) | BD Biosciences |
| TIGIT (clone IG9) | Biolegend |
| ef-506 | eBioscience |
| Lag3 (clone C9B7W) | BD Biosciences |
| GITR (clone DTA-1) | eBioscience |
| CTLA-4 (clone UC10-4B9) | Biolegend |
| OX-40 (clone OX-86) | Biolegend |
| NK 1.1 (clone PK136) | Biolegend |
| Ter119 | Fluidigm |
| CD122 (clone TMB1) | BD Biosciences |
| CD27 (clone LG.3A10) | BD Biosciences |
| CD69 (clone H1.2F3) | BD Biosciences |
| CD8a (clone 53-6.7) | BD Biosciences |
| CX3CR1 (SA011F11) | Biolegend |
| CD127 (A7R34) | Miltenyi Biotec |
| CD49b (clone DX5) | BD Biosciences |
| CD161 (clone PK136) | Biolegend |
| CD3e (clone 145-2C11) | eBioscience |
| CD25 (clone PC61) | BD Biosciences |
| CCR7 (clone 4B12) | Biolegend |
| CD62L (clone MEL14) | BD Biosciences |

**Mass cytometry**

| CD11b (clone M1/70) | Biolegend |
| --- | --- |
| CD4 (clone GK1.5) | Biolegend |
| CD39 (clone 24DMS1) | Fluidigm |
| Lag3 (clone C9B7W) | Biolegend |
| IL-2 (clone JES6-5H4) | Fluidigm |
| CD69 (clone H1.2F3) | Fluidigm |
| CD8a (clone 53-6.7) | Fluidigm |
| CD62L (clone MEL-14) | Biolegend |
| CD335 (clone 29A1.4) | Biolegend |
| IFNy (clone XMG1.2) | Biolegend |
| CD27 (clone LG.3A10) | Fluidigm |
| CD25 (clone 3C7) | Fluidigm |
| CD3e (clone 145-2C11) | Fluidigm |
| CD366 (clone B8.2C12) | Biolegend |
| CD152 (clone UC10-4B9) | Fluidigm |
| CD279 (clone 29F.1A12) | Biolegend |
| CD226 (clone 480.1) | Biolegend |
| IL-10 (clone JES5-16E3) | Fluidigm |
| Rorgt (clone B2D) | Fluidigm |
| Tbet (clone 4B10) | Fluidigm |
| NKG2AE (clone 20d5) | BD Biosciences |
| Ki-67 (clone B56) | Fluidigm |
| Klrg1 (clone 2F1) | eBioscience |
| TIGIT (clone 1G9) | Biolegend |
| FoxP3 (clone FJK-16s) | Fluidigm |
| IL-4 (clone 11B11) | Fluidigm |
| IL-6 (clone MP5-20F3) | Fluidigm |
| Helios (clone 22F6) | Biolegend |
| TCRβ (clone H57-597) | Fluidigm |
| CD154 (clone MR1) | Fluidigm |
| CD44 (clone IM7) | Fluidigm |
| Perforin (clone OMAK-D) | Fluidigm |
| GranzymeB (clone GB11) | Fluidigm |
| IL-17A (clone TC11-18H10.1) | Fluidigm |
| Gata3 (clone 16E10A23) | Biolegend |
| CD45R (clone RA3-6B2) | Fluidigm |
| CD45.2 (clone 104) | Biolegend |
